# Induced Treg Cells Augment the Th17-Mediated Intestinal Inflammatory Response in a CTLA4-Dependent Manner

**DOI:** 10.1371/journal.pone.0150244

**Published:** 2016-03-07

**Authors:** Nobumasa Watanabe, Osamu Kaminuma, Noriko Kitamura, Takachika Hiroi

**Affiliations:** Allergy and Immunology Project, Tokyo Metropolitan Institute of Medical Science, Tokyo, Japan; Mie University Graduate School of Medicine, JAPAN

## Abstract

Th17 cells and Foxp3^+^ regulatory T cells (Tregs) are thought to promote and suppress inflammatory responses, respectively. However, whether they counteract each other or synergize in regulating immune reactions remains controversial. To determine their interactions, we describe the results of experiments employing mouse models of intestinal inflammation by transferring antigen-specific Th cells (Th1, Th2, and Th17) differentiated *in vitro* followed by the administration of the cognate antigen via enema. We show that cotransfer of induced Tregs (iTregs) suppressed Th1- and Th2-mediated colon inflammation. In contrast, colon inflammation induced by transfer of Th17 cells, was augmented by the cotransfer of iTregs. Furthermore, oral delivery of antigen potentiated Th17-mediated colon inflammation. Administration of a blocking antibody against cytotoxic T lymphocyte-associated antigen 4 (CTLA4) abrogated the effects of cotransfer of iTregs, while the injection of a soluble recombinant immunoglobulin (Ig) fusion protein, CTLA4-Ig substituted for the cotransfer of iTregs. These results suggest that antigen-specific activation of iTregs in a local environment stimulates the Th17-mediated inflammatory response in a CTLA4-dependent manner.

## Introduction

Accumulating evidence indicates that CD4^+^ helper T cells play a central role in eliciting normal immune responses and in inducing inappropriate reactions leading to allergy and autoimmune diseases [1]. For example, CD4^+^ regulatory T cells (Tregs) that express the transcription factor FoxP3 represent a distinct cell population with immunnosuppressive function [1–3]. In contrast, effector CD4^+^ helper T cells are classified mainly into Th1, Th2, and Th17 subsets that induce physiological immune responses depending on the infectious pathogens. Unless attenuated after elimination of pathogens, or maintained tolerance to self or innocuous antigens, activation of these effector subsets initiates allergic or inflammatory disorders. The idea that an aberrant Th2-type immune response induces allergy and is regulated by FoxP3^+^ Tregs is consistent with the results of studies on humans and numerous mouse models [4–6]. In contrast, the pathogenic role of Th17 cells on the development of autoimmune and inflammatory disorders remains controversial although the vast majority of recent findings from genome-wide studies of humans and mouse models support the intimate involvement of this subset in promoting the diseases [7–9]. This ambiguity may be explained as follows. First, most studies employ mouse models, including spontaneous occurrence of the diseases, which are driven by combinations of various T cell subsets, resembling human disease [10], which impedes the evaluation of the contribution of Th17 cells to pathogenesis. Second, the properties of Th17 cells are diverse and highly plastic in terms of immunological functions, including immune suppression under certain conditions [11–13]. Therefore, whether Th17-type immunity is susceptible to immunological tolerance or suppression mediated by FoxP3^+^ Tregs remains largely unknown. Moreover, evidence indicates that Tregs support the development of Th17 cells or promote Th17-mediated immunological responses [14–18] by secreting TGF-beta [19] or by consumption of IL-2 [17, 18]. Irrespective of the outcomes of interactions between Th17 cells and Tregs, the role of antigen specificity must be considered. Therefore, to delineate the outcomes caused by one-to-one interactions between iTregs and each effector T cells from otherwise complex immunological responses, we employed a model in which antigen-specific CD4^+^ T cells are adoptively transferred in combination followed by antigen delivery. We show here that the differential effects of iTregs depending on the effector subsets, and that CTLA4 is critically involved in both processes, inhibition of Th1/Th2-mediated colon inflammation and stimulation of Th17-mediated colon inflammation.

## Results and Discussion

### Antigen-specific effector cells induce colon thickening

**Fig 1:**
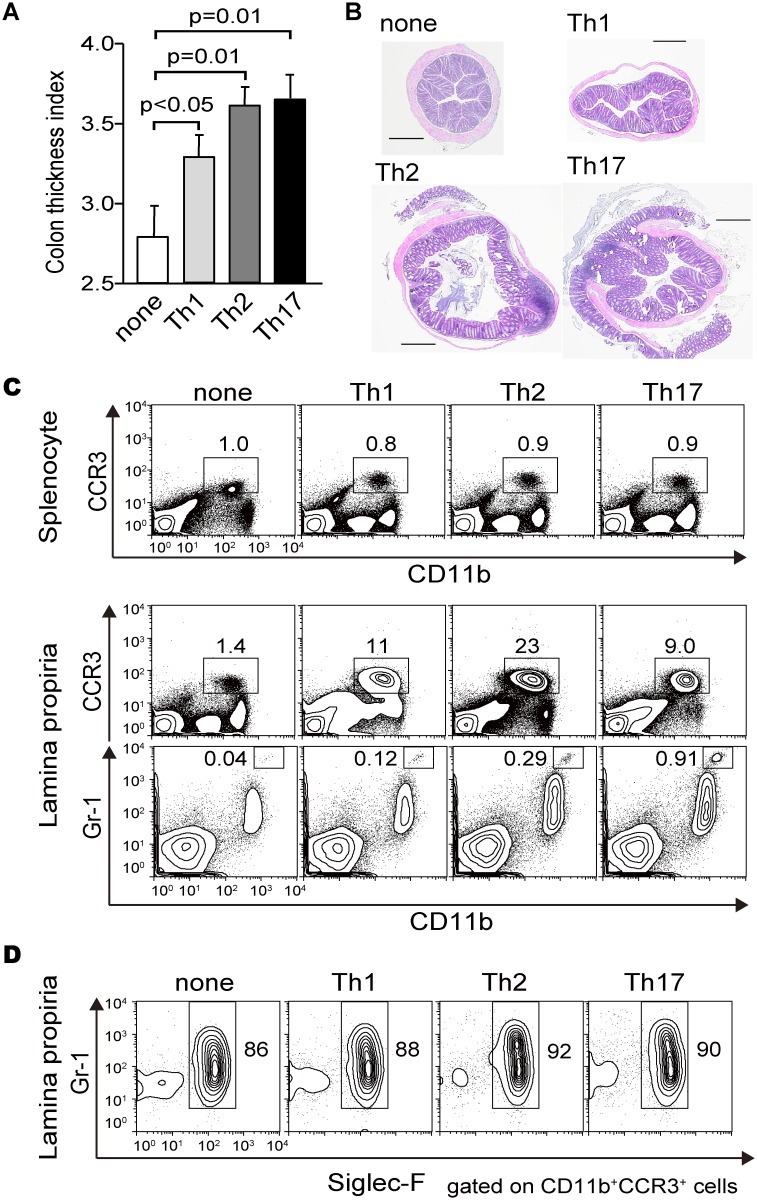
Adoptive transfer of effector T cells induces colon thickening. Each effector T cell subset (2 × 10^7^ viable cells) was intravenously transferred to wild-type BALB/c mice, and OVA protein was administered via enema once a week for 4 weeks. The day after the last OVA challenge, the colonic weight-to-length ratio (mg/mm) was calculated as the colon thickness index (CTI) to evaluate the inflammatory response. (A) Three types of effector cells (Th1/Th2/Th17) induced CTI using this model mouse. (B) Representative histological images of HE-stained mid-colonic sections are shown. Scale bars indicate 500 microm. (C) Mononuclear cells were isolated from the spleen and cLP of recipient mice. CD11b^+^ CCR3^+^ and CD11b^+^ Gr-1^+^ cells were gated, and their frequencies (%) were determined using flow cytometric analysis. Representative data of three independent experiments are shown. (D) Mononuclear cells were isolated from the cLP of recipient mice and stained with monoclonal antibodies (mAbs) against CD11b, CCR3, Gr-1, and Siglec-F. The frequencies (%) of Siglec-F^+^ Gr-1^middle^ cells in the total population of CD11b^+^ CCR3^+^ cells are shown

CD4^+^ T cells were obtained from spleen and mesenteric lymph nodes of DO11.10 transgenic mice with a *Rag2*-deficient background (DO11.10^+^:Rag2 KO). Cells were differentiated in vitro in a mutually exclusive manner to an interferon (IFN)-gamma-producing Th1 subset, an interleukin (IL)-4-producing Th2 subset, and an IL-17A-producing Th17 subset under each polarizing condition described in Materials and Methods (S1A Fig) [20]. Approximately 2 × 10^7^ viable cells of each subset were intravenously transferred to wild-type BALB/c mice, and ovalbumin (OVA) was administered via enema once a week for 4 weeks. One day after the last administration of OVA, colon length and weight were measured and the length-to-weight ratio (colon thickness index, CTI), which correlates with histological scores [21], was calculated. Regardless of the Th subset (Th1, Th2, Th17) derived in vitro, all effector subsets increased CTI values, suggesting that each possesses intrinsic activities that induce an inflammatory response in the intestinal tract (Fig 1A and 1B). Furthermore, the induction of colon thickening depended on transfer of cells and delivery of antigen (S2 Fig).

Accumulation of eosinophils defined as CCR3^+^CD11b^+^ mononuclear cells in the colon lamina propria (cLP) was more prominent in mice that received Th2 cells (Fig 1C), which is consistent with the increased production of IL-5 (S1B Fig). CCR3^+^CD11b^+^ populations specifically expressed the eosinophil marker Siglec-F (Fig 1D). In contrast, the number of CD11b^+^Gr-1^+^ cells, which are thought to be neutrophils involved in Th17-type immunity [22, 23], were slightly increased in the cLP of mice that received Th17 cells compared with those in the cLP of mice that received Th1 and Th2 cells (Fig 1C).

### Antigen-specific iTregs stimulate Th17-mediated colon thickening

**Fig 2:**
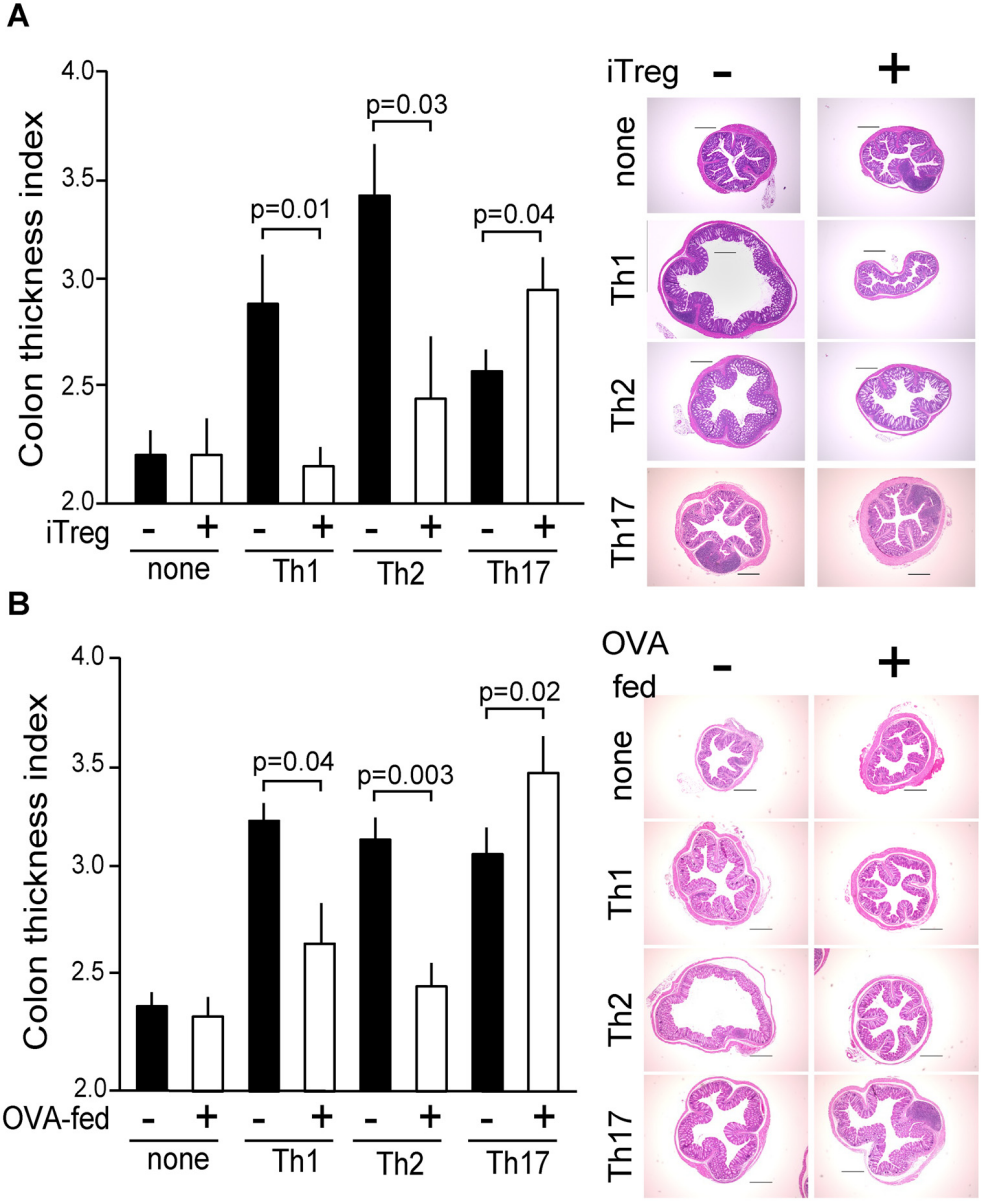
Co-transfer of iTregs or oral administration of OVA stimulates colon thickening mediated by Th17. (A) Each effector cell (2 × 10^7^ cells per mouse: Th1, Th2, Th17) was adoptively transferred or not (none) with or without iTregs (1 × 10^7^ cells per mouse) into wild-type BALB/c mice and each mouse was immunized with OVA as described in Fig 1. CTI values are shown as the mean and standard error (SE). Independent experimental sets were designed for histological analysis, and representative images are shown. (B) Before adoptive transfer, BALB/c mice were continuously supplied with OVA (1 mg/mL) in their drinking water for 7 days to induce oral tolerance (indicated as OVA-fed). Each effector cell (2 × 10^7^ cells/mouse: Th1/Th2/Th17) was adoptively transferred, and mice were treated as described in Fig 1. CTI and histological analysis were performed as described above.

Next, we examined the susceptibility of effector subsets to the suppressive function of iTregs that recognized the same epitope. For this purpose, OVA323-339 epitope-specific iTregs that expressed FoxP3 predominantly and increased levels of CD25 and CTLA4 were prepared (S1A Fig). Data acquired using similar models, that employ cotransfer of antigen-specific iTregs, followed by the administration of an antigen, regardless of target organ such as the respiratory or digestive tract [24–26], suggest that Th2 cells are highly susceptible to iTregs, however, the susceptibility of Th17 cells to the cotransfer of iTregs has been an arguable issue [27]. We show here that iTregs almost completely suppressed Th1 and Th2 cell-mediated colon thickening, although transfer of iTregs alone had no effect on CTI (Fig 2A), consistent with previous reports [24–26]. In contrast, simultaneously prepared iTregs that suppressed Th1 and Th2 cell-mediated colon thickening, stimulated Th17-mediated colon thickening. Accumulating evidence indicates that lineage stability of iTregs in vivo is not as robust as expected [28]. Therefore it might be possible that iTregs co-transferred with Th17 cells, but not with Th1 or Th2 cells may convert to Th17-like population with some effector function. This issue remains to be clarified further.

### Oral-administration of OVA stimulates Th17-mediated colon thickening

Oral administration of antigen suppresses not only gut mucosal but also systemic immune responses, particularly Th2-type allergic reactions against challenge with the same antigen (oral tolerance) [29–31]. Moreover, the function(s) of FoxP3^+^ Tregs are prerequisite for establishing oral tolerance to allergic immune reactions such as allergic diarrhea and ear swelling induced by protein antigens [32]. However, it remains unknown whether immune responses derived solely from Th17 cells are affected by oral tolerization. We addressed this issue considering the stimulatory effects of iTregs on the Th17-mediated colon thickening described above. We applied a standard protocol to induce oral tolerance, by providing a continuous supply of OVA in drinking water for 7 days. This procedure resulted in the increased FoxP3^+^ ratio of transferred naïve DO^+^T cells not only prepared from cLP but also from spleen (S3A Fig). However, FoxP3^+^ ratio of endogenous CD4^+^T cells was unaltered, presumably because of the scarcity of the OVA-specific CD4^+^T cells detectable in this analysis (S3B Fig). Consistent with conventional findings, Th2- and Th1-mediated colon thickening were highly susceptible to immune suppression by prior oral administration of OVA, although the Th17-mediated increase in CTI was not inhibited but accelerated by tolerization (Fig 2B). Furthermore, histological analysis indicated the contrasting effects of oral tolerance on Th1/Th2- and Th17-mediated pathology.

### Colon thickening is induced less efficiently by IL17A-deficient Th17 cells

**Fig 3:**
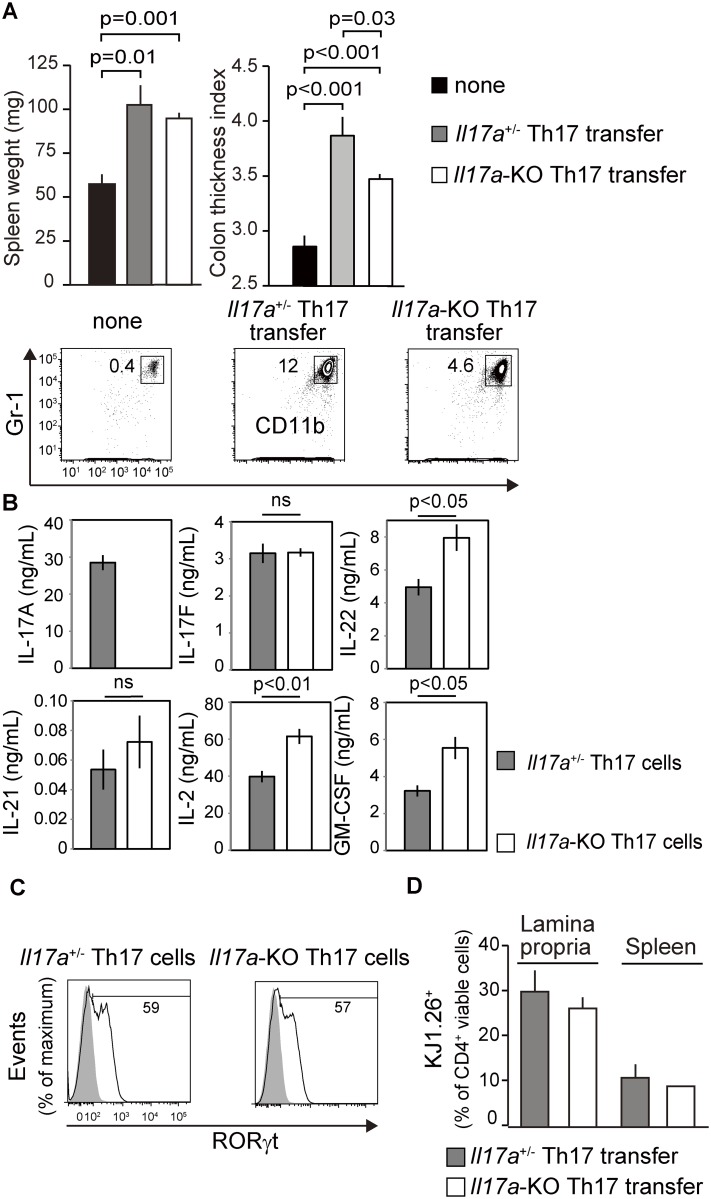
*Il17a*-deficient Th17 cells induce a diminished but significant inflammatory responses. (A) Th17 cells were differentiated in vitro from naïve CD4^+^T cells derived from *Il17a*-deficient (*Il17a*-KO:DO11.10^+^:*Rag2*-KO) or *Il17a*-sufficient (*Il17a*^+/−^:DO11.10^+^:*Rag2*-KO) mice. Eosinophil-deficient deltadblGATA mice were engrafted and treated with OVA as described above. Spleen weights were measured and analyzed (*n* = 4). The weight-to-length ratio of the colon was calculated and expressed as CTI. Mononuclear cells of the cLP were prepared and subjected to flow cytometric analysis to determine the frequencies of CD11b^+^ Gr-1^+^ cells. Representative flow cytometry data of two separately performed and reproducibly repeated experiments are shown. (B) *Il17a*^+/−^ Th17 or *Il17a*-KO Th17 cells were restimulated using the anti-CD3epsilon-/anti-CD28-conjugated beads (Life Technologies) for 48 h. Secreted cytokines were quantified using ELISA, as described in Materials and Methods. (C) Restimulated cells were subjected to flow cytometric analysis to investigate RORgammat expression as a marker of Th17 cells. The shaded histogram shows the control experiment using an isotype-matched antibody. Frequencies of RORgammat^+^ cells are indicated. (D) deltadblGATA mice were engrafted with *Il17a*^+/−^ Th17 or *Il17a*-KO Th17 cells and immunized as described in Fig 1. Mononuclear cells were prepared from the cLP and spleen and stained with mAbs against CD3epsilon, CD4, and DO11.10 TCR. The frequencies of DO11.10 TCR^+^ cells in the total population of CD3^+^CD4^+^ cells are shown.

IL-17A, which is expressed specifically by Th17 cells, plays a pivotal role in Th17-type immune responses, particularly in the recruitment of neutrophils to inflamed sites [33]. In fact, there are a couple of reports showing that IL-17A deficiency abrogated the immunopathology driven by Th17 cells [34]. Therefore, we determined the relative contribution of IL-17A to the Th17-mediated immune response in our experimental settings. For this purpose, we employed eosinophil-deficient mice as recipients to evaluate the accumulation of CD11b^+^Gr-1^+^ cells in the cLP in addition to the induction of colon thickening. Th17 cells derived and differentiated from IL17A-deficient mice mediated these processes, although to a diminished but significant extent (Fig 3A). Splenomegaly was present not only in mice engrafted with IL17A-sufficient Th17 cells but also in mice engrafted with IL17A-defficient Th17 cells (Fig 3A). The expression of IL-17F, which is most closely related to IL-17A, and the lineage-restricted transcription factor RORgammat were comparable to those of IL17A-sufficient Th17 cells in vitro (Figs 2, 3B and 3C). Moreover, migration to the cLP and the probability of survival were unaltered in vivo (Fig 3D). Therefore, the induction of colon thickening and accumulation of CD11b^+^Gr-1^+^ cells by engrafting Th17 cells may be due to the redundant functional sums of the activities of IL-17A and other Th17-related cytokines. In fact, a couple of cytokines other than IL-17F, which are reported to be involved in Th17-mediated pathology, were more expressed in IL17A-deficient Th17 cells at least in vitro (Fig 3B), suggesting the intrinsic effect of IL-17A on the cytokine profile produced by Th17 cells. Thus the pathology via transferring IL17A-deficient Th17 cells might be driven by these cytokines.

### Cotransfer of iTregs inhibits Th2-mediated colon thickening and stimulates Th17-mediated colon thickening using eosinophil-deficient mice as recipients

**Fig 4:**
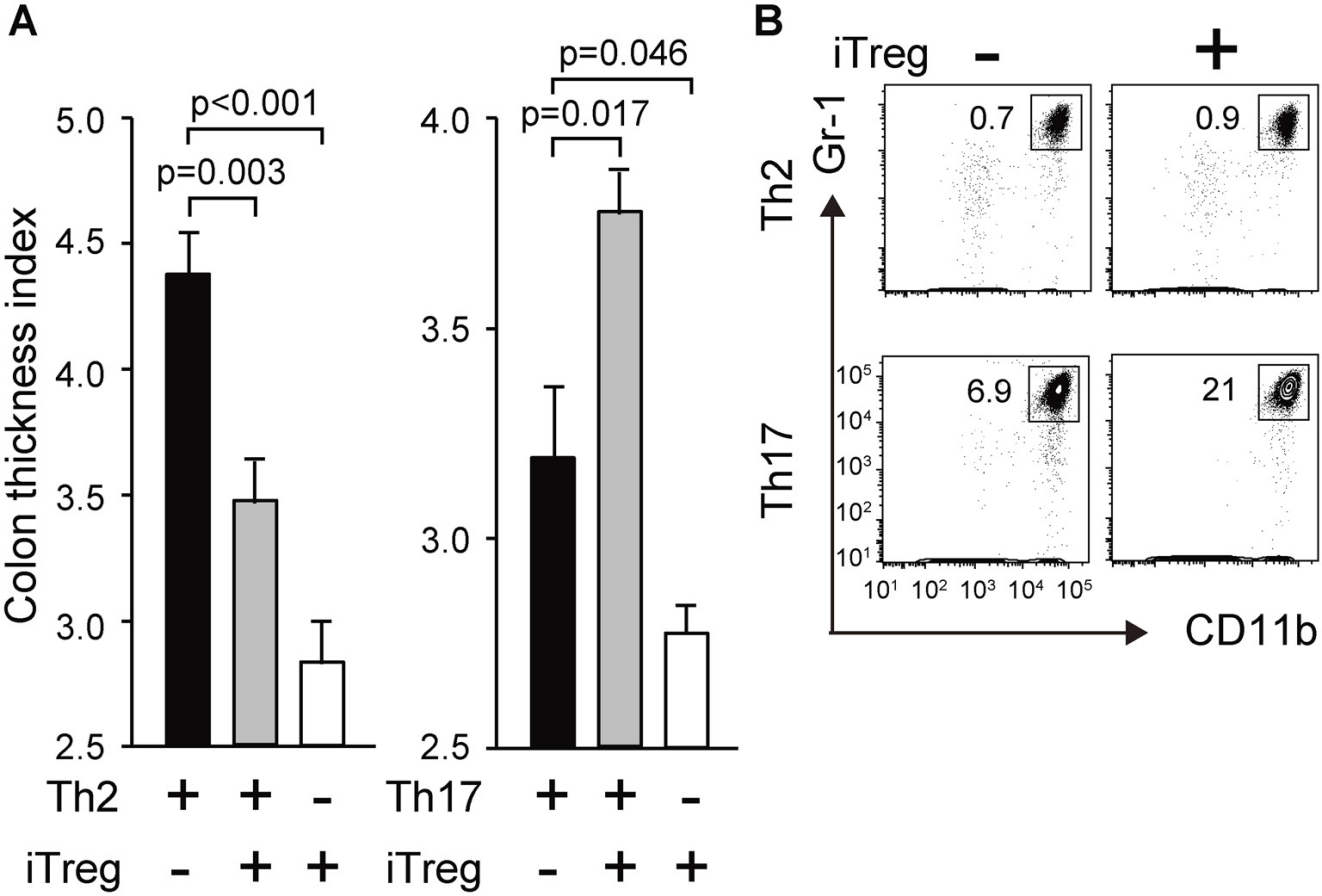
Antigen-specific iTregs exacerbate Th17-mediated colon inflammation in eosinophil-deficient mice. deltadblGATA mice were adoptively engrafted with effector T cells (2×10^7^ cells /mouse: Th2/Th17) alone or together with iTregs (1 × 10^7^ cells/mouse). (A) Recipient mice were challenged with OVA and CTI values were determined. (B) Cells were prepared from the cLP and subjected to flow cytometric analysis to determine the percentage of neutrophils. Representative flow cytometry data of two independent experiments are shown.

Transfer of effector T cells, particularly Th2 cells, led to the accumulation of eosinophils in the cLP, which is considered an inflammatory response of the intestinal tract (Fig 1). Therefore, we attempted to evaluate the involvement of eosinophils in the induction of CTI using eosinophil-deficient mice as recipients. Both Th17 cells and Th2 cells induced colon thickening in eosinophil-deficient recipients (Fig 4A), indicating that eosinophils residing in the cLP were dispensable for the induction of CTI or that other granulocytic populations may compensate in eosinophil-deficient mice. Moreover, the accumulation of CD11b^+^Gr-1^+^ cells was increased in eosinophil-deficient mice engrafted with Th17, but not Th2 cells (Fig 4B). These observations indicate that the accumulation of neither eosinophils nor neutrophils directly leads to colon thickening, although subset-specific immune reactions may occur. In contrast, differential effects of iTregs on Th2 and Th17 cell-mediated colon thickening were reconfirmed using eosinophil-deficient mice as recipients (Fig 4A). Furthermore, CD11b^+^Gr-1^+^ cells were much more abundant in mice that received iTreg and Th17 cells together compared with those that received Th17 cells alone (Fig 4B), indicating that iTregs play a dominant role in determining the stimulatory or inhibitory effect on colon thickening, irrespective of the type of granulocytes residing in the cLP.

Several kinds of molecular apparatus have been proposed to account for the immunomodulatory function of FoxP3^+^ Tregs [2, 35]. Of these, we tested the contribution of IL-10 expressed intrinsically in CD4^+^ T cell to colon thickening. The *Il10*-deficiency of Th2 cells induced a hyper-Th2 phenotype, that produced massive amounts of Th2-related cytokines in vitro (S4A and S4B Fig) and a higher abundance of eosinophils in the cLP in vivo (S4C Fig). However, *Il10*-deficient iTregs suppressed the colon thickening mediated by *Il10*-deficient Th2 cells with efficiency comparable to that of *Il10*-sufficient iTregs (S4D Fig). The expression levels of FoxP3, CD25, and CTLA4 in iTregs differentiated in vitro were equivalent, irrespective of the presence of *Il10* (S4E Fig). Therefore, we next focused on the role of CTLA4 in this model system.

### Anti-CTLA4 antibody abrogates the effects of iTregs and a CTLA4-Ig fusion protein mimics iTreg function

**Fig 5:**
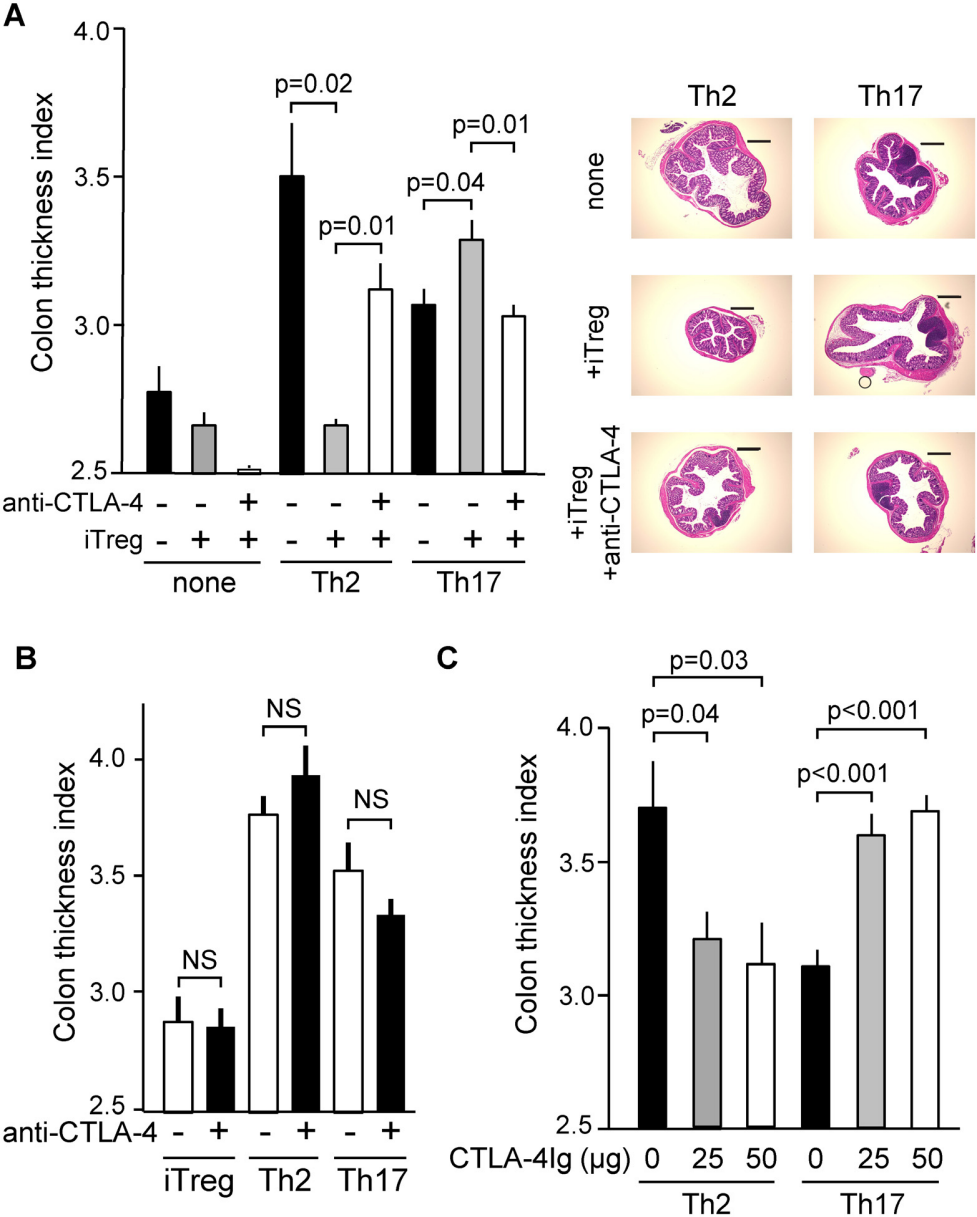
Anti-CTLA4 antibody abrogates the effects of iTregs, and soluble CTLA4 alone mimics the effects of iTregs. (A) Effector T cells (Th2 or Th17) were intravenously transferred with or without iTregs in the presence or absence of anti-CTLA4 antibody (20 microg) and mice were treated as described in Fig 1. CTI values were calculated and are shown as mean and standard error (SE). Independent experimental sets were designed for histological analysis, and representative images are shown. (B) iTregs or effector T cells (Th2 or Th17) were intravenously transferred in the presence of anti-CTLA4 antibody (20 microg) or control antibody (20 microg, indicated as minus) and mice were treated as described in Fig 1. (C) Effector T cells (Th2 or Th17) were intravenously transferred in the presence or absence of an indicated amount of CTLA4-Ig, and mice were treated as described in Fig 1. CTI values were calculated and are shown as mean and SE.

Although effector T cells other than Tregs express CTLA4 after stimulation [36], FoxP3^+^ cell-restricted deletion of *Ctla4* leads to a sub-lethal multifocal inflammatory disorder similar to that caused by systemic deletion of *Ctla4*, albeit with a later age of onset [37]. This finding indicates that FoxP3^+^ Tregs require CTLA4 to restrain immune responses. We first employed an anti-CTLA4 antibody to evaluate the contribution of CTLA4 to iTreg-mediated modulation of colon thickening. Administration of the anti-CTLA4 antibody simultaneously cell transfer abrogated the effects of the iTregs, i.e., suppression of Th2-mediated colon thickening and enhancement of Th17-mediated colon thickening (Fig 5A). Histological observations were consistent with CTI values. It might be possible that anti-CTLA4 antibody directly targets Th17 cells or Th2 cells since CTLA4 is induced after activation of the effector T cells. However, administration of anti-CTLA4 antibody does not have significant effect on Th2- or Th17-mediated CTI induction in the absence of iTregs (Fig 5B). In addition, anti-CTLA4 antibody shows no effect on viability of engrafted iTregs or status of FoxP3 expression in vivo (S5 Fig). Therefore, it is likely that anti-CTLA4 antibody hampers the function of CTLA4 expressed on iTregs as well as, if any, endogenous Tregs. In this context, we were intrigued that FoxP3^+^ cell-specific deletion of *Ctla4* resulted in an increase of the number of IFN-gamma^+^ or IL-4^+^ cells, but not that of IL-17^+^ cells [37], suggesting CTLA4 expressed on FoxP3^+^ cells plays a less prominent role in regulating the Th17-type response, yet apparent functional role in suppressing Th1- and Th2-type immune responses. Furthermore, deletion of *Stat3* from FoxP3^+^ cells induces hyperactivation of Th17 cells in vivo, while *Stat3*-deficient Tregs express higher levels of *Ctla4* mRNA compared with *Stat3*-sufficient Tregs do [38], suggesting that CTLA4 expressed by FoxP3^+^ cells may have little effect on suppressing Th17-type immunity or otherwise have a function to promote Th17-type immunity.

In contrast, recent reports show that Th17 cells acquired a follicular helper T cell (T_FH_ cell) phenotype in vivo to induce an antigen-specific IgA production [39] or ectopic lymphoid follicles [40]. Consistent with these findings, transfer of Th17 cells increased OVA-specific IgA production, although Th1 and Th2 cells exhibited enhancement of OVA-specific IgA production compared with the control (S6A Fig). Th17-driven OVA-specific IgA secretion, however, was diminished by cotransfer of iTregs and abrogated again by simultaneous administration of the anti-CTLA4 antibody (S6B Fig). This suggests that the versatile functions of Th17 cells in vivo are distinct in terms of susceptibility to the iTreg-CTLA4 axis.

**Fig 6:**
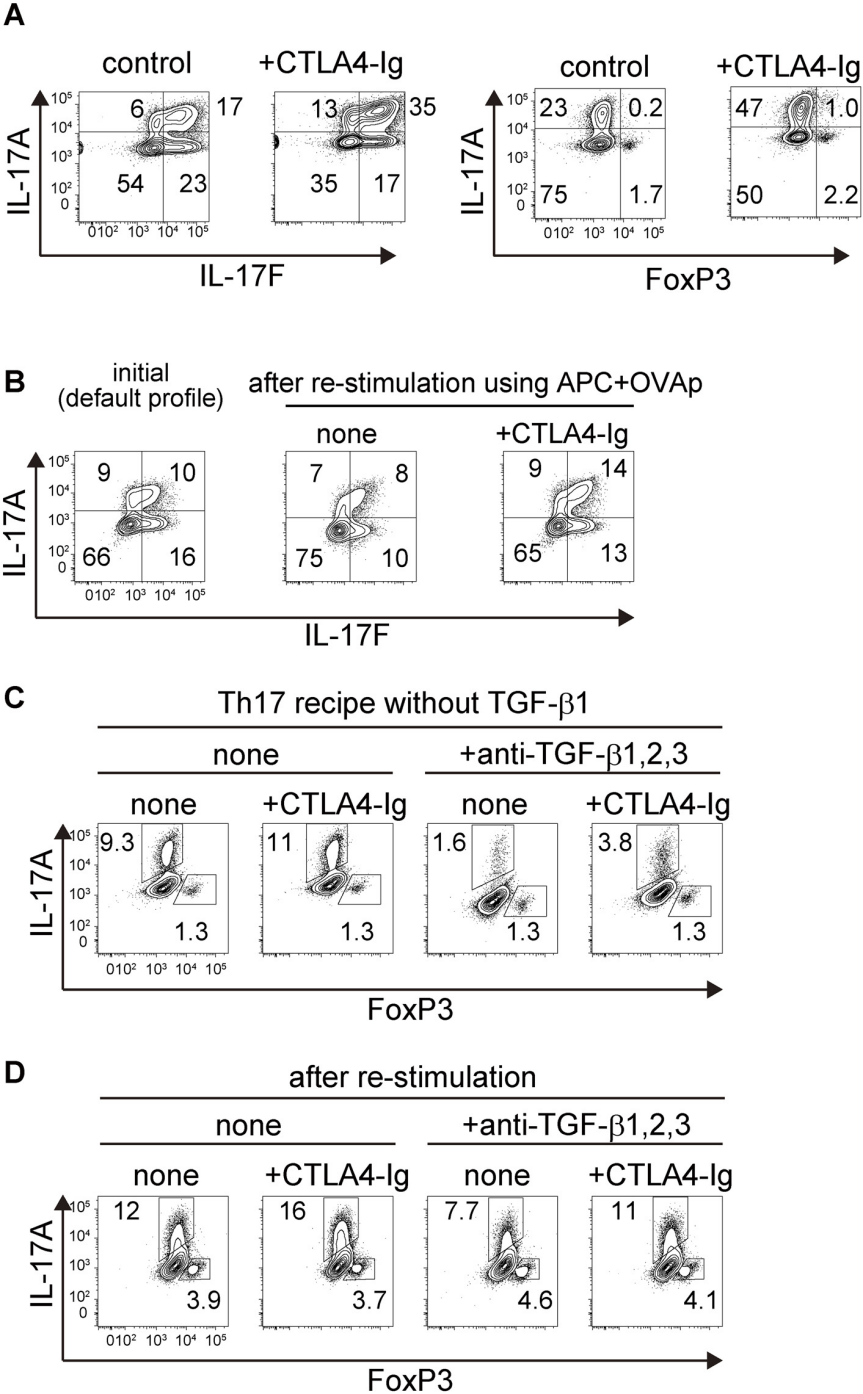
CTLA4-Ig augments the ratio of IL-17A-producing cells in the presence of anti-TGF-beta antibody. (A) CD4^+^ T cells prepared from DO11.10^+^:*Rag2*-KO mice were stimulated under the condition for Th17 cell lineage, namely medium supplemented with IL-6, IL-23, TGF-beta1, IL-1beta and TNF-alpha, in the absence (control) or presence of CTLA4-Ig (+CTLA4-Ig, 20microg/mL). After 7 days, cells were restimulated with PMA (20 ng /mL) and ionomycin (1 μM) in the presence of monensin for 4 h. Cells were stained with anti-CD4 and treated with FVD and subjected to the analysis for intracellular expression of the indicated cytokines (IL-17A and IL-17F) and FoxP3. (B) Th17 cells were stimulated again using the APCs (irradiated splenocytes derived from *Rag2*-KO mice) and OVA peptide in the medium without cytokines in the absence (control) or presence of CTLA4-Ig (+CTLA4-Ig, 20microg/mL). After 6 days, cells were examined as described in (A). (C) Th17 cells were differentiated using the medium supplement with IL-6, IL-23 IL-1beta and TNF-alpha, but not with TGF-beta1 (indicated as Th17 recipe without TGF-beta1), in the absence (none) or presence (+anti-TGF-beta1,2,3, 10microg/mL) of anti-TGF-beta antibody in conjunction with CTLA4-Ig (20 microg/mL) or not. After 6 days, cells were examined as described in (A). (D) Th17 cells were re-stimulated as described in (B) in the absence (none) or presence (+anti-TGF-beta1,2,3) of anti-TGF-beta antibody in conjunction with CTLA4-Ig or not as shown in (C). After 6 days, cells were examined as described in (A).

CTLA4 is regarded as an immunosuppressive molecule and numerous studies elucidating its molecular action [41, 42] have advanced the development of therapeutic applications for various immune-related disorders as well as anti-tumor immunotherapy [36, 43]. For example, the soluble fusion protein CTLA4-immunoglobulin (CTLA4-Ig) suppresses immune reactions in vitro and in vivo, presumably by binding to costimulatory ligands expressed by antigen presenting cells to block CD28 signaling in effector T cells [44]. In fact, CTLA4-Ig is effectively used to treat autoimmune and inflammatory diseases such as rheumatoid arthritis [45, 46]. Therefore, we addressed the effect of CTLA4-Ig on Th2- or Th17-driven intestinal immune responses. Th2 and Th17-mediated colon thickening responded differentially to the administration of CTLA4-Ig, in other words, Th2-mediated colon thickening was inhibited and at the same time and doses, Th17-mediated colon thickening was accelerated (Fig 5C). In addition, the frequency of DO^+^Th17 cells, but not of DO^+^Th2 cells among CD4^+^T cells was slightly increased in mice administered CTLA4-Ig (S7 Fig). These results indicate that restriction of costimulatory ligand availability and following inhibition of CD28 signaling, both of which are induced by CTLA4-Ig, lead to the opposite outcomes depending on the effector T cell subsets. This finding seems to be compatible with that of study showing that the differentiation of Th17 cells is blocked by an anti-CD28 antibody, indicating the adverse effect of CD28 signaling on the development of Th17 cells, at least in vitro [47]. In fact, we showed that differentiation of DO^+^Th17 cells as well as iTregs from naïve CD4+T cells was accelerated in the presence of CTLA4-Ig in terms of IL-17A^+^ ratio and FoxP3^+^ ratio, respectively (Fig 6A and S8 Fig), which is consistent with a report mentioned above [47]. Moreover, we found that even in the re-stimulation phase the addition of CTLA4-Ig maintains or rather augments the frequency of IL-17A-producing cells (Fig 6B). These results might indicate that administration of CTLA4-Ig favor the differentiation of Th17 cells through curbing of CD28 signaling, which is reminiscent of the function of TGF-beta in this process [48]. In this context, we examined the effect of anti-TGF-beta antibody in conjunction with CTLA4-Ig in our in vitro system. As a result, the addition of CTLA4-Ig increased the frequency of IL17A-producing cells even in the presence of anti-TGF-beta antibody in both process, differentiation from naïve to Th17 cells (Fig 6C) and re-stimulation of Th17 cells (Fig 6D). These observations seem to be consistent with the scenario that both immunomodulatory molecules, CTLA4 and TGF-beta act independently on the cell surface but following events merge to inhibit CD28-signaling cascade within the cells.

In this context, we were intrigued that mice administered an agonistic anti-CD3epsilonantibody, which induces TCR stimulation without CD28 signaling, shows preferential accumulation of IL-17A-producing CD4^+^ T cells in the small intestine [11]. In the aggregate, Th17-type immune responses may favor the signal input delivered via the T-cell receptor under conditions of costimulation blockade. Therefore, targeting the CTLA4/CD28 signaling pathway should be carefully evaluated in relation to the presence of pathogenic effector subsets and target organs. In this respect, it is worth to note that in phase III trials, CTLA4-Ig had no significant benefit for patients with inflammatory bowel diseases and, on the contrary, exacerbated ulcerative colitis [49]. Moreover, it was reported that patients with rheumatoid arthritis developed ulcerative colitis during the treatment of CTLA4-Ig [50, 51].

The relevance of Th17-driven colon inflammatory responses described in the present study must be considered from the perspective of the pathogenesis of inflammatory bowel disease in the future. Taken together, the results of the present study demand reconsideration of Treg/CTLA4-based immunological modulation to suppress or treat autoimmune diseases, particularly in patients with Th17-driven intestinal inflammation.

## Materials and Methods

### Mice

Balb/c mice were purchased from Nihon SLC (Shizuoka, Japan), DO11.10×*Rag2*-KO mice, *Il10*-KO mice, and eosinophil-deficient (deltadblGATA) mice were obtained from The Jackson Laboratory (Bar Harbor, ME). *Il17A*-KO mice were provided by Dr. Iwakura. *Il10*-KO mice and *Il17A*-KO mice were backcrossed with Balb/c genetic background more than 10 times in our facility to produce *Il10*-KO or *Il17A*-KO×DO11.10×*Rag2*-KO mice. Female mice 8 to 12 weeks older were used and bred in specific pathogen-free facilities at the Tokyo Metropolitan Institute of Medical Science. This study was carried out in strict accordance with the guidelines in the Proper Conduct of Animal Experiments, as defined by the Science Council of Japan and the Animal Care and Use Committees of the Tokyo Metropolitan Institute of Medical Science approved all experimental procedures (Permit Number:15035and 15036).

### Evaluation of colon thickening and histology

The colon, starting from just below the cecum to above the anal, was excised. Connective tissues were removed, cut longitudinally, washed to remove fecal material, following which length and weight were measured to calculate the length-to-weight ratio. Independent experimental groups were used to obtain transverse sections of mid-to distal-colons. for histology. Specimens were formalin-fixed, embedded in paraffin, sectioned, and stained with hematoxylin and eosin.

### Reagents

We purchased OVA from Sigma-Aldrich (St Louis, MO) and OVA peptide (323–339) from Scrum Inc. (Tokyo, Japan).

### Antibodies

eFluor 450-CD11b (M1/70;), APC-eFluor 780-Gr-1 (RB6-8C5), FITC- and Pacific Blue-IFN-gamma (XMG1.2), PE-Cy7-IL-4 (BVD6-24G2), Alexa Fluor 647- and Alexa Fluor 488-IL-17A (eBio17B7), PerCP-Cy5.5-FoxP3 (FJK-16s), PE-RORgammat (B2D), PE-IL-10 (JES5-16E3), APC-eFluor 780-CD4 (RM4-5), purified CD16/32 (93), and 7-AAD were purchased from eBioscience. We purchased fluorescein-CCR3 (#83101) from R&D Systems; PE-Cy5-CD45 (30-F11), PE-Siglec-F (E50-2440), PE-CD152 (UC10-4F10-11), and PE-DO11.10 (KJ1-26, BD) from BD Biosciences; Alexa Fluor 488-CD25 (PC61) and Alexa Fluor 647-IL-17F (9D3.1C8) from BioLegend; and VioBlue-DO11.10 (KJ1-26) from Miltenyi Biotec. Purified anti-CD152(CTLA4) antibody (9H10) and isotype control antibody (SHG-1) used for in vivo studies was purchased from BioLegend, and anti-TGF-beta1,2,3 antibody (1D11) was R&D systems. The soluble fusion protein of CTLA4 and immunoglobulin (Ig) G1 Fc region (CTLA4-Ig, Abatacept) was purchased from Bristol-Myers Squibb.

### *In vitro* differentiation and adoptive transfer of OVA-specific T cells

Antigen-specific effector T cells were prepared as described previously [20]. Approximately 2 × 10^7^ viable effector T cells were transferred intravenously with or without 1 × 10^7^ viable iTregs.

### OVA Treatment

Two hundred microliters of OVA solution (10 mg/mL dissolved in PBS) was injected intra-rectally with animal feeding needles (1.5 mm od × 52-mm long, FUCHIGAMI, Kyoto, Japan), such that the tip was 4 cm proximal to the anus. This treatment was repeated five times daily at approximately 10 min intervals for each. To establish oral tolerance, mice were fed with drinking water supplemented with 1 mg/mL OVA for 7 days before adoptive transfer of cells.

### Quantification of cytokines and OVA-specific IgA

Cells were activated using Dynabeads T-Activator CD3/CD28 (Life Technologies) or by culturing with antigen-presenting cells (irradiated splenocytes derived from *Rag2*-KO) and OVA (50 mg/mL), and cytokines in the supernatants were measured using ELISA kits for IL-17F, IL-22, IL-21 and GM-CSF (eBioscience) or a multiplex bead array for IL-5, IL-13, IL-4, IL-10, IL-17A, and IL-2 (Millipore). Fecal extracts were obtained by adding weighed pellets to PBS (1 mL/100 mg fecal sample) containing protease inhibitors (P8340; Sigma-Aldrich). The samples were mixed and centrifuged, and the supernatants were collected for assay. OVA-specific IgA titers were determined using an ELISA with OVA as the capture antigen, and immune complexes were detected using horseradish peroxidase-conjugated anti-mouse IgA (Southern Biotech).

### Preparation of cells from the cLP and flow cytometric analysis

Cells from the cLP were prepared by cutting the large intestine into 1-cm long pieces, and then stirred for 20 min at 37°C in PBS containing 5 mM EDTA and 5 mM EGTA to dissociate epithelial and intraepithelial cells. After washing with PBS three times, the remaining tissue was treated for 50 min at 37°C with RPMI containing 2 mg/mL collagenase D (Roche) and 1 mg/mL DNase I (Roche). Mononuclear cells were isolated using a discontinuous Percoll gradient (40% and 75%) and subjected to flow cytometry (FACScantoII, BD Biosciences). Following stimulation with PMA (20 ng/mL) and ionomycin (1μM) in the presence of monensin for 4h, intracellular staining of the cells was performed using Foxp3 Fixation/Permeabilization Concentrate and Diluent (eBioscience) according to the manufacturer’s instructions.

### Statistical analysis

Data were analyzed using an unpaired two-tailed Student’s *t* test. A P-values of <0.05 was considered statistically significant.

## Supporting Information

S1 Fig Flow cytometric profiles of T cell subsets (Th1, Th2, Th17, and iTreg cells). CD4^+^T cells prepared from DO11.10^+^:*Rag2*-KO mice were polarized under conditions appropriate for each T cell lineage in the presence of APCs and OVA323-339 peptide (0.3microM), as described in Materials and Methods. (A) After 7 days, the cultured cells were restimulated with PMA (20 ng /mL) and ionomycin (1 μM) in the presence of monensin for 4 h. Cells were reacted with anti-CD4, anti-DO11.10 TCR, and anti-CD25 antibodies and treated with Fixable Viability Dye (FVD). The detection of intracellular expression of the indicated cytokines, transcription factors (FoxP3 and RORgammat), and CTLA4 is described in Materials and Methods. CD4^+^DO11.10 TCR^+^ FVD^−^ cells were gated for the analysis. (B) Cells (1 × 10^6^) were stimulated in the presence of APCs (1 × 10^6^, irradiated splenocytes derived from *Rag2*-KO mice) and OVA (50 microg/mL) for 48 h and cytokine levels in culture supernatants were measured using multiplex bead assay.
(TIF)

S2 Fig Specificity of colon thickness induction. Wild-type mice were engrafted or not engrafted with *Il10*-deficient Th2 cells and were not treated (none), or challenged with (OVA), or with (BSA) via enema.
(TIF)

S3 Fig Administration of OVA increased the frequencies of FoxP3^+^ cells among the transferred DO^+^CD4^+^ cells. (A) CD4^+^T cells (1 × 10^7^) prepared from DO11.10^+^:*Rag2*-KO mice were transferred to wild-type BALB/c mice, and OVA protein was administered in a drinking water (1mg/mL) for a week (OVA-fed) or not (control). Mononuclear cells (MNCs) were isolated from the spleen (SPL) and colon lamina propria (cLP) of mice and subjected to the flow cytometric analysis. Frequencies of CD4^+^DO (KJ1.26)^+^ cells were shown and gated populations were analyzed for FoxP3 expression and ratio of FoxP3^+^ cells were shown in histograms. (B) Wild-type BALB/c mice were treated as described in (A). MNCs were isolated from the spleen (SPL) and subjected to the flow cytometric analysis as described in (A).
(TIF)

S4 Fig *Il10*-deficient iTregs repress Th2-mediated colon thickening. Th2 and iTregs were differentiated in vitro from CD4^+^ T cells derived from *Il10*-deficient (*Il10*-KO:DO11.10^+^:*Rag2*-KO) or *Il10*-sufficient (*Il10*^+/+^:DO11.10^+^:*Rag2*-KO) mice. (A) *Il10*-sufficient (*Il10*-WT) and *Il10*-deficient (*Il10*-KO) Th2 cells were restimulated with 12-*O*-Tetradecanoylphorbol-13-acetate (PMA) (20 ng /mL) and ionomycin (1 μM) in the presence of monensin for 4 h. Cells were stained with mAbs against CD4, and the T cell receptor (DO11.10), and treated with Fixable Viability Dye, and subjected to flow cytometric analysis after incubation with antibodies against IL-4 and IL-10. The frequencies of cells expressing IL-4 or IL-10 were determined according to populations gated on CD4^+^DO11.10 TCR^+^ cells. (B) *Il10*-WT and *Il10*-KO Th2 cells were restimulated for 48 h in vitro using the anti-CD3epsilon-/anti-CD28-conjugated beads, and secreted cytokines were quantified as described in Materials and Methods. All experiments were reproducibly repeated at least twice, and a representative data set is shown. (C) *Il10*-WT Th2 cells or *Il10*-KO Th2 cells along with *Il10*-sufficient iTregs or *Il10*-deficient iTregs were transferred to wild-type BALB/c mice and mice. Mononuclear cells (MNCs) were isolated from the cLP of mice engrafted with each combination of cells as indicated and incubated with antibodies against CCR3 and Siglec-F. Representative flow cytometric profiles are shown with the frequencies of CCR3^+^Siglec-F^+^ cells in MNCs isolated from the cLP. (D) CTI values were calculated and are shown as mean and standard error (SE). (E) *Il10*-sufficient (*Il10*-WT) and *Il10*-deficient (*Il10*-KO) iTregs were restimulated as in (A) and stained with anti-CD4, anti-DO11.10 TCR, and anti-CD25 mAbs followed by Fixable Viability Dye staining. Cells were subjected to flow cytometry to determine the intracellular expression of IL-4, IL-10, CTLA4, and FoxP3. CD4^+^DO11.10 TCR^+^ FVD^−^ cells were gated for the analysis.
(TIF)

S5 Fig Anti-CTLA4 antibody shows no effect on survival of iTregs. (A) MNCs were isolated from the spleen and cLP of mice engrafted with iTregs in the presence of anti-CTLA4 antibody (20 microg) or control antibody (20 microg) and treated as described in Fig 1. CD4^+^ T cells were enriched using anti-CD4 magnetic beads (Miltenyi Biotech) and stained with indicated antibodies. Representative flow cytometric profiles are shown with the frequencies of CD3epsilon^+^DO^+^ cells and FoxP3^+^ cells gated on CD3epsilon^+^DO^+^ cells. (B) As a control for FoxP3 staining, Th17 cells were used and analyzed in the same way as described in (A).
(TIF)

S6 Fig Increase of IgA secretion by Th17 cells is suppressed by cotransfer of iTregs. (A) Fecal extracts were prepared the day after the third challenge with OVA, and OVA-specific IgA was detected using ELISA to estimate relative endpoint titers as described in Materials and Methods. (B) Th17 cells with or without iTregs were transferred in the absence or presence of an anti-CTLA4 antibody (20 microg). After the third challenge with OVA, fecal samples were collected, and relative titers of OVA-specific IgA were determined.
(TIF)

S7 Fig The effect of CTLA4-Ig on probability of survival of Th17 cells and Th2 cells *in vivo*. Effector T cells (Th2 or Th17) were intravenously transferred in the presence or absence of CTLA4-Ig (50microg), and mice were treated as described in Fig 1. MNCs were isolated from the SPL or cLP of mice engrafted and subjected to the flow cytometric analysis. Frequencies of CD3epsilon^+^CD4^+^ cells were shown and gated populations were analyzed for KJ1.26 staining and ratio of DO (KJ1.26)^+^ cells were shown in histograms.
(TIF)

S8 Fig CTLA4-Ig augments the ratio of FoxP3^+^ cells. CD4^+^ T cells prepared from DO11.10^+^:*Rag2*-KO mice were stimulated under the condition for iTreg lineage, namely medium supplemented with IL-2, TGF-beta1 and retinoic acid (iTreg recipe), or medium containing IL-2 and TGF-beta1 but not retinoic acid (iTreg recipe without retinoic acid) in the absence (control) or presence of CTLA4-Ig (+CTLA4-Ig, 20microg/mL). After 7 days, cells were subjected to the analysis as described in S1 Fig.
(TIF)

## References

[1] Campbell DJ, Koch MA. Phenotypical and functional specialization of FOXP3+ regulatory T cells. Nat Rev Immunol. 2011;11(2):119–30. Epub 2011/01/27. 10.1038/nri2916 21267013 PMC3289970

[2] Sakaguchi S, Yamaguchi T, Nomura T, Ono M. Regulatory T cells and immune tolerance. Cell. 2008;133(5):775–87. Epub 2008/05/31. 10.1016/j.cell.2008.05.009 18510923

[3] Wing K, Sakaguchi S. Regulatory T cells exert checks and balances on self tolerance and autoimmunity. Nat Immunol. 2010;11(1):7–13. Epub 2009/12/18. 10.1038/ni.1818 . 20016504

[4] Tian L, Altin JA, Makaroff LE, Franckaert D, Cook MC, Goodnow CC, et al Foxp3(+) regulatory T cells exert asymmetric control over murine helper responses by inducing Th2 cell apoptosis. Blood. 2011;118(7):1845–53. Epub 2011/07/01. 10.1182/blood-2011-04-346056 21715314 PMC3158716

[5] Curotto de Lafaille MA, Kutchukhidze N, Shen S, Ding Y, Yee H, Lafaille JJ. Adaptive Foxp3+ regulatory T cell-dependent and -independent control of allergic inflammation. Immunity. 2008;29(1):114–26. Epub 2008/07/12. 10.1016/j.immuni.2008.05.010 . 18617425

[6] Josefowicz SZ, Niec RE, Kim HY, Treuting P, Chinen T, Zheng Y, et al Extrathymically generated regulatory T cells control mucosal TH2 inflammation. Nature. 2012;482(7385):395–9. Epub 2012/02/10. 10.1038/nature10772 22318520 PMC3485072

[7] Franke A, McGovern DP, Barrett JC, Wang K, Radford-Smith GL, Ahmad T, et al Genome-wide meta-analysis increases to 71 the number of confirmed Crohn's disease susceptibility loci. Nat Genet. 2010;42(12):1118–25. Epub 2010/11/26. 10.1038/ng.717 21102463 PMC3299551

[8] Annunziato F, Cosmi L, Santarlasci V, Maggi L, Liotta F, Mazzinghi B, et al Phenotypic and functional features of human Th17 cells. J Exp Med. 2007;204(8):1849–61. Epub 2007/07/20. 10.1084/jem.20070663 17635957 PMC2118657

[9] Littman DR, Rudensky AY. Th17 and regulatory T cells in mediating and restraining inflammation. Cell. 2010;140(6):845–58. Epub 2010/03/23. 10.1016/j.cell.2010.02.021 . 20303875

[10] Neurath MF, Finotto S. Translating inflammatory bowel disease research into clinical medicine. Immunity. 2009;31(3):357–61. Epub 2009/09/22. 10.1016/j.immuni.2009.08.016 . 19766078

[11] Esplugues E, Huber S, Gagliani N, Hauser AE, Town T, Wan YY, et al Control of TH17 cells occurs in the small intestine. Nature. 2011;475(7357):514–8. Epub 2011/07/19. 10.1038/nature10228 21765430 PMC3148838

[12] Chalmin F, Mignot G, Bruchard M, Chevriaux A, Vegran F, Hichami A, et al Stat3 and Gfi-1 transcription factors control Th17 cell immunosuppressive activity via the regulation of ectonucleotidase expression. Immunity. 2012;36(3):362–73. Epub 2012/03/13. 10.1016/j.immuni.2011.12.019 . 22406269

[13] Muranski P, Restifo NP. Essentials of Th17 cell commitment and plasticity. Blood. 2013;121(13):2402–14. Epub 2013/01/18. 10.1182/blood-2012-09-378653 23325835 PMC3612853

[14] Vokaer B, Van Rompaey N, Lemaitre PH, Lhomme F, Kubjak C, Benghiat FS, et al Critical role of regulatory T cells in Th17-mediated minor antigen-disparate rejection. J Immunol. 2010;185(6):3417–25. Epub 2010/08/25. 10.4049/jimmunol.0903961 . 20733201

[15] Moore-Connors JM, Fraser R, Halperin SA, Wang J. CD4(+)CD25(+)Foxp3(+) regulatory T cells promote Th17 responses and genital tract inflammation upon intracellular Chlamydia muridarum infection. J Immunol. 2013;191(6):3430–9. Epub 2013/08/21. 10.4049/jimmunol.1301136 . 23956419

[16] Vokaer B, Charbonnier LM, Lemaitre PH, Spilleboudt C, Le Moine A. IL-17A and IL-2-expanded regulatory T cells cooperate to inhibit Th1-mediated rejection of MHC II disparate skin grafts. PLoS One. 2013;8(10):e76040 Epub 2013/10/23. 10.1371/journal.pone.0076040 24146810 PMC3795694

[17] Chen Y, Haines CJ, Gutcher I, Hochweller K, Blumenschein WM, McClanahan T, et al Foxp3(+) regulatory T cells promote T helper 17 cell development in vivo through regulation of interleukin-2. Immunity. 2011;34(3):409–21. Epub 2011/03/26. 10.1016/j.immuni.2011.02.011 . 21435588

[18] Pandiyan P, Conti HR, Zheng L, Peterson AC, Mathern DR, Hernandez-Santos N, et al CD4(+)CD25(+)Foxp3(+) regulatory T cells promote Th17 cells in vitro and enhance host resistance in mouse Candida albicans Th17 cell infection model. Immunity. 2011;34(3):422–34. Epub 2011/03/26. 10.1016/j.immuni.2011.03.002 21435589 PMC3258585

[19] Veldhoen M, Hocking RJ, Atkins CJ, Locksley RM, Stockinger B. TGFbeta in the context of an inflammatory cytokine milieu supports de novo differentiation of IL-17-producing T cells. Immunity. 2006;24(2):179–89. Epub 2006/02/14. 10.1016/j.immuni.2006.01.001 . 16473830

[20] Kaminuma O, Ohtomo T, Mori A, Nagakubo D, Hieshima K, Ohmachi Y, et al Selective down-regulation of Th2 cell-mediated airway inflammation in mice by pharmacological intervention of CCR4. Clin Exp Allergy. 2012;42(2):315–25. Epub 2011/11/19. . 22092376 10.1111/j.1365-2222.2011.03847.x

[21] Ostanin DV, Bao J, Koboziev I, Gray L, Robinson-Jackson SA, Kosloski-Davidson M, et al T cell transfer model of chronic colitis: concepts, considerations, and tricks of the trade. Am J Physiol Gastrointest Liver Physiol. 2009;296(2):G135–46. Epub 2008/11/27. 10.1152/ajpgi.90462.2008 19033538 PMC2643911

[22] van de Veerdonk FL, Gresnigt MS, Kullberg BJ, van der Meer JW, Joosten LA, Netea MG. Th17 responses and host defense against microorganisms: an overview. BMB Rep. 2009;42(12):776–87. Epub 2010/01/05. . 20044948 10.5483/bmbrep.2009.42.12.776

[23] Chang SH, Mirabolfathinejad SG, Katta H, Cumpian AM, Gong L, Caetano MS, et al T helper 17 cells play a critical pathogenic role in lung cancer. Proc Natl Acad Sci U S A. 2014;111(15):5664–9. Epub 2014/04/08. 10.1073/pnas.1319051111 24706787 PMC3992670

[24] Huter EN, Stummvoll GH, DiPaolo RJ, Glass DD, Shevach EM. Cutting edge: antigen-specific TGF beta-induced regulatory T cells suppress Th17-mediated autoimmune disease. J Immunol. 2008;181(12):8209–13. Epub 2008/12/04. 19050237 10.4049/jimmunol.181.12.8209 PMC2788513

[25] Stummvoll GH, DiPaolo RJ, Huter EN, Davidson TS, Glass D, Ward JM, et al Th1, Th2, and Th17 effector T cell-induced autoimmune gastritis differs in pathological pattern and in susceptibility to suppression by regulatory T cells. J Immunol. 2008;181(3):1908–16. Epub 2008/07/22. 18641328 10.4049/jimmunol.181.3.1908 PMC2561289

[26] Girtsman T, Jaffar Z, Ferrini M, Shaw P, Roberts K. Natural Foxp3(+) regulatory T cells inhibit Th2 polarization but are biased toward suppression of Th17-driven lung inflammation. J Leukoc Biol. 2010;88(3):537–46. Epub 2010/05/25. 10.1189/jlb.0110044 20495073 PMC2924601

[27] Chen X, Oppenheim JJ. Th17 cells and Tregs: unlikely allies. J Leukoc Biol. 2014 Epub 2014/02/25. 10.1189/jlb.1213633 24563509 PMC3984971

[28] Zhou L, Chong MM, Littman DR. Plasticity of CD4+ T cell lineage differentiation. Immunity. 2009;30(5):646–55. Epub 2009/05/26. 10.1016/j.immuni.2009.05.001 . 19464987

[29] Tsuji NM, Kosaka A. Oral tolerance: intestinal homeostasis and antigen-specific regulatory T cells. Trends Immunol. 2008;29(11):532–40. Epub 2008/10/07. 10.1016/j.it.2008.09.002 . 18835746

[30] Mayer L, Shao L. Therapeutic potential of oral tolerance. Nat Rev Immunol. 2004;4(6):407–19. Epub 2004/06/03. 10.1038/nri1370 . 15173830

[31] Pabst O, Mowat AM. Oral tolerance to food protein. Mucosal Immunol. 2012;5(3):232–9. Epub 2012/02/10. 10.1038/mi.2012.4 22318493 PMC3328017

[32] Hadis U, Wahl B, Schulz O, Hardtke-Wolenski M, Schippers A, Wagner N, et al Intestinal tolerance requires gut homing and expansion of FoxP3+ regulatory T cells in the lamina propria. Immunity. 2011;34(2):237–46. Epub 2011/02/22. 10.1016/j.immuni.2011.01.016 . 21333554

[33] Iwakura Y, Ishigame H, Saijo S, Nakae S. Functional specialization of interleukin-17 family members. Immunity. 2011;34(2):149–62. Epub 2011/02/26. 10.1016/j.immuni.2011.02.012 . 21349428

[34] Zhu S, Qian Y. IL-17/IL-17 receptor system in autoimmune disease: mechanisms and therapeutic potential. Clin Sci (Lond). 2012;122(11):487–511. Epub 2012/02/14. 10.1042/CS20110496 . 22324470

[35] Wing JB, Sakaguchi S. Foxp3+ Treg cells in humoral immunity. Int Immunol. 2013 Epub 2013/12/11. 10.1093/intimm/dxt060 . 24324208 PMC7108613

[36] Walker LS, Sansom DM. The emerging role of CTLA4 as a cell-extrinsic regulator of T cell responses. Nat Rev Immunol. 2011;11(12):852–63. Epub 2011/11/26. 10.1038/nri3108 . 22116087

[37] Wing K, Onishi Y, Prieto-Martin P, Yamaguchi T, Miyara M, Fehervari Z, et al CTLA-4 control over Foxp3+ regulatory T cell function. Science. 2008;322(5899):271–5. Epub 2008/10/11. 10.1126/science.1160062 . 18845758

[38] Chaudhry A, Rudra D, Treuting P, Samstein RM, Liang Y, Kas A, et al CD4+ regulatory T cells control TH17 responses in a Stat3-dependent manner. Science. 2009;326(5955):986–91. Epub 2009/10/03. 10.1126/science.1172702 . 19797626 PMC4408196

[39] Hirota K, Turner JE, Villa M, Duarte JH, Demengeot J, Steinmetz OM, et al Plasticity of Th17 cells in Peyer's patches is responsible for the induction of T cell-dependent IgA responses. Nat Immunol. 2013;14(4):372–9. Epub 2013/03/12. 10.1038/ni.2552 23475182 PMC3672955

[40] Peters A, Pitcher LA, Sullivan JM, Mitsdoerffer M, Acton SE, Franz B, et al Th17 cells induce ectopic lymphoid follicles in central nervous system tissue inflammation. Immunity. 2011;35(6):986–96. Epub 2011/12/20. 10.1016/j.immuni.2011.10.015 22177922 PMC3422678

[41] Onishi Y, Fehervari Z, Yamaguchi T, Sakaguchi S. Foxp3+ natural regulatory T cells preferentially form aggregates on dendritic cells in vitro and actively inhibit their maturation. Proc Natl Acad Sci U S A. 2008;105(29):10113–8. Epub 2008/07/19. 10.1073/pnas.0711106105 18635688 PMC2481354

[42] Qureshi OS, Zheng Y, Nakamura K, Attridge K, Manzotti C, Schmidt EM, et al Trans-endocytosis of CD80 and CD86: a molecular basis for the cell-extrinsic function of CTLA-4. Science. 2011;332(6029):600–3. Epub 2011/04/09. 10.1126/science.1202947 21474713 PMC3198051

[43] Shevach EM. Mechanisms of foxp3+ T regulatory cell-mediated suppression. Immunity. 2009;30(5):636–45. Epub 2009/05/26. 10.1016/j.immuni.2009.04.010 . 19464986

[44] Linsley PS, Wallace PM, Johnson J, Gibson MG, Greene JL, Ledbetter JA, et al Immunosuppression in vivo by a soluble form of the CTLA-4 T cell activation molecule. Science. 1992;257(5071):792–5. Epub 1992/08/07. . 1496399 10.1126/science.1496399

[45] Rosenblum MD, Gratz IK, Paw JS, Abbas AK. Treating human autoimmunity: current practice and future prospects. Sci Transl Med. 2012;4(125):125sr1 Epub 2012/03/17. 10.1126/scitranslmed.3003504 . 22422994 PMC4061980

[46] Moreland L, Bate G, Kirkpatrick P. Abatacept. Nat Rev Drug Discov. 2006;5(3):185–6. Epub 2006/03/25. 10.1038/nrd1989 . 16557658

[47] Bouguermouh S, Fortin G, Baba N, Rubio M, Sarfati M. CD28 co-stimulation down regulates Th17 development. PLoS One. 2009;4(3):e5087 Epub 2009/04/01. 10.1371/journal.pone.0005087 19333372 PMC2658739

[48] Delisle JS, Giroux M, Boucher G, Landry JR, Hardy MP, Lemieux S, et al The TGF-beta-Smad3 pathway inhibits CD28-dependent cell growth and proliferation of CD4 T cells. Genes Immun. 2013;14(2):115–26. Epub 2013/01/19. 10.1038/gene.2012.63 . 23328844

[49] Sandborn WJ, Colombel JF, Sands BE, Rutgeerts P, Targan SR, Panaccione R, et al Abatacept for Crohn's disease and ulcerative colitis. Gastroenterology. 2012;143(1):62–9.e4. Epub 2012/04/17. 10.1053/j.gastro.2012.04.010 . 22504093

[50] Amezcua-Guerra LM, Hernandez-Martinez B, Pineda C, Bojalil R. Ulcerative colitis during CTLA-4Ig therapy in a patient with rheumatoid arthritis. Gut. 2006;55(7):1059–60. Epub 2006/06/13. 10.1136/gut.2006.095539 16766771 PMC1856333

[51] Motohashi R, Ikeuchi H, Hiromura K, Ohishi Y, Sakurai N, Sakairi T, et al Two cases of ulcerative colitis developing in rheumatoid arthritis patients during abatacept therapy. Scand J Gastroenterol. 2014;49(10):1270–1. Epub 2014/08/15. 10.3109/00365521.2014.946087 . 25115461

