# Supplementary figures and images for: Induced Treg Cells Augment the Th17-Mediated Intestinal Inflammatory Response in a CTLA4-Dependent Manner
Source: PLoS One. 2016 Mar 7;11(3):e0150244. doi: 10.1371/journal.pone.0150244 (PMC4780716; doi:10.1371/journal.pone.0150244)

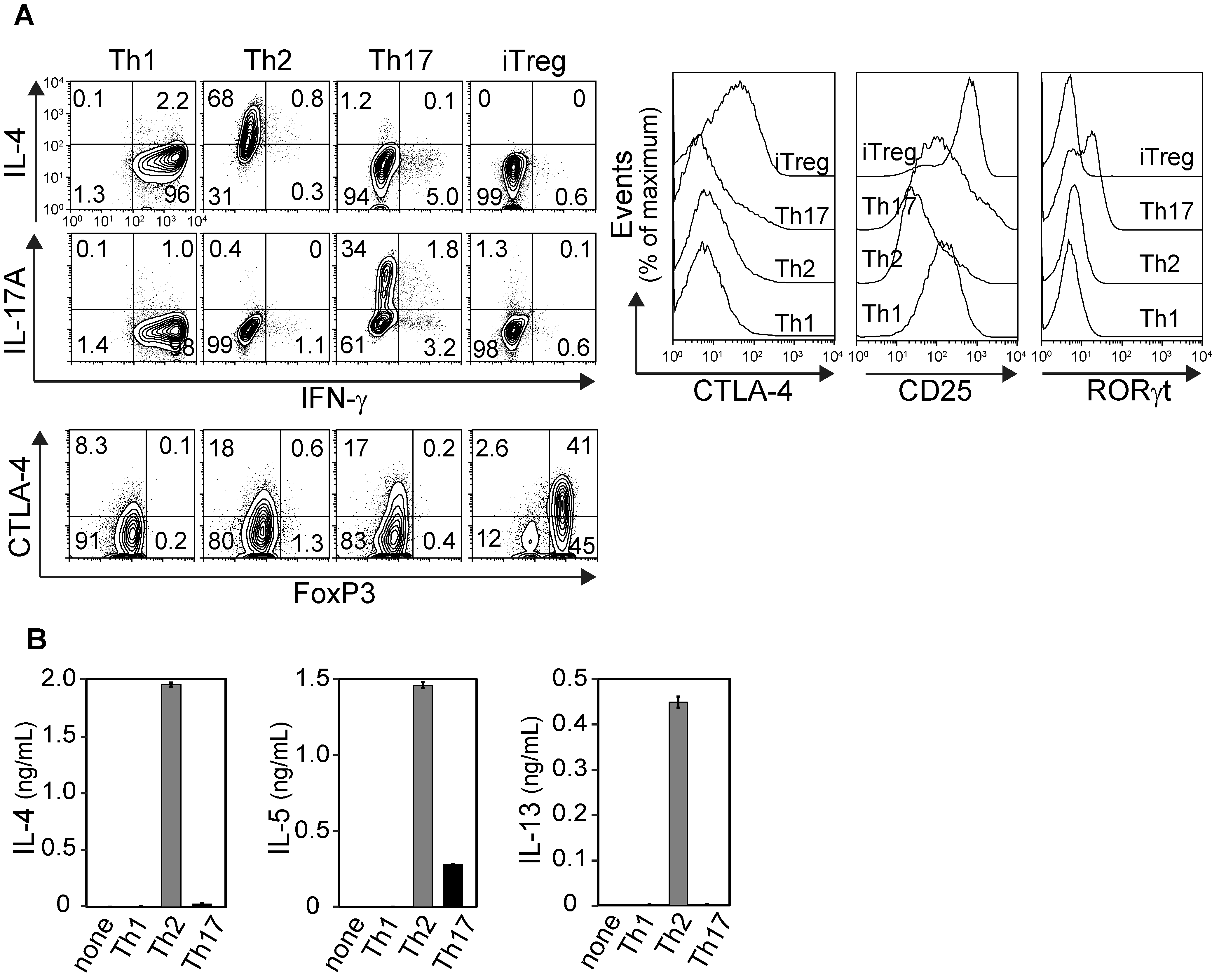

Supplement: S1 Fig — CD4+T cells prepared from DO11.10+:Rag2-KO mice were polarized under conditions appropriate for each T cell lineage in the presence of APCs and OVA323-339 peptide (0.3microM), as described in Materials and Methods. (A) After 7 days, the cultured cells were restimulated with PMA (20 ng /mL) and ionomycin (1 μM) in the presence of monensin for 4 h. Cells were reacted with anti-CD4, anti-DO11.10 TCR, and anti-CD25 antibodies and treated with Fixable Viability Dye (FVD). The detection of intracellular expression of the indicated cytokines, transcription factors (FoxP3 and RORgammat), and CTLA4 is described in Materials and Methods. CD4+DO11.10 TCR+ FVD− cells were gated for the analysis. (B) Cells (1 × 10^6^) were stimulated in the presence of APCs (1 × 10^6^, irradiated splenocytes derived from Rag2-KO mice) and OVA (50 microg/mL) for 48 h and cytokine levels in culture supernatants were measured using multiplex bead assay. (TIF) [file pone.0150244.s001.tif]

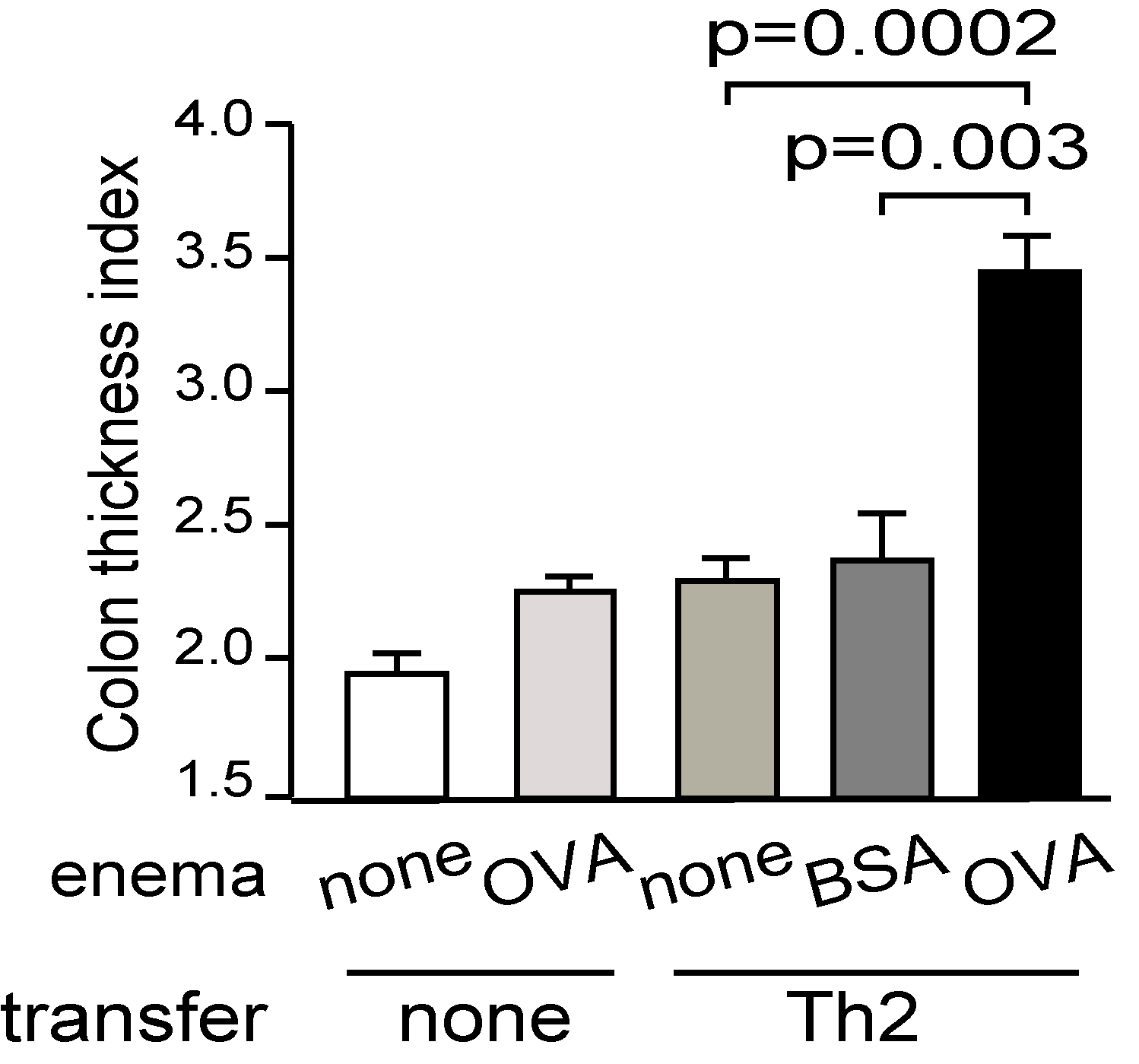

Supplement: S2 Fig — Wild-type mice were engrafted or not engrafted with Il10-deficient Th2 cells and were not treated (none), or challenged with (OVA), or with (BSA) via enema. (TIF) [file pone.0150244.s002.tif]

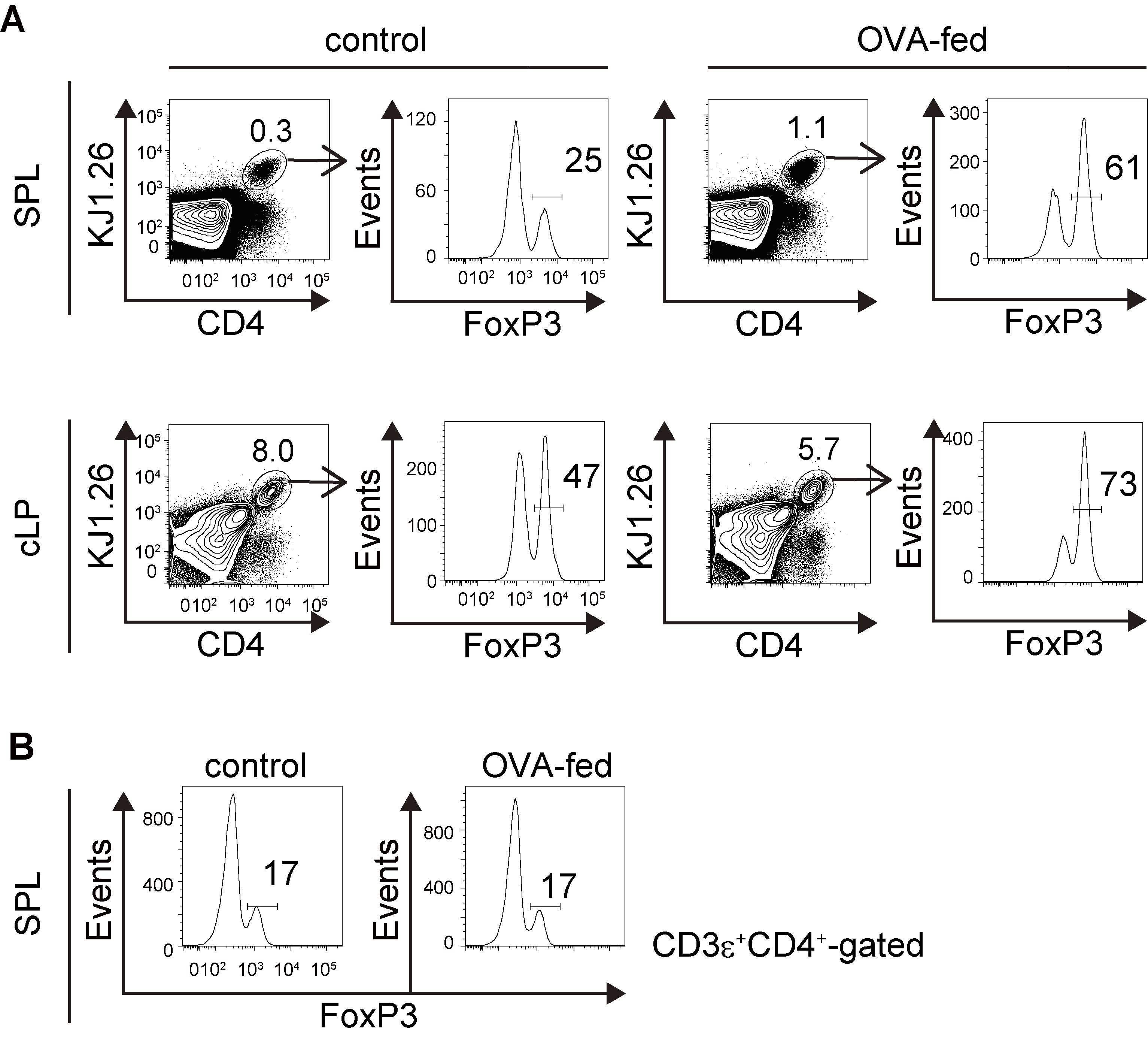

Supplement: S3 Fig — (A) CD4+T cells (1 × 10^7^) prepared from DO11.10+:Rag2-KO mice were transferred to wild-type BALB/c mice, and OVA protein was administered in a drinking water (1mg/mL) for a week (OVA-fed) or not (control). Mononuclear cells (MNCs) were isolated from the spleen (SPL) and colon lamina propria (cLP) of mice and subjected to the flow cytometric analysis. Frequencies of CD4+DO (KJ1.26)+ cells were shown and gated populations were analyzed for FoxP3 expression and ratio of FoxP3+ cells were shown in histograms. (B) Wild-type BALB/c mice were treated as described in (A). MNCs were isolated from the spleen (SPL) and subjected to the flow cytometric analysis as described in (A). (TIF) [file pone.0150244.s003.tif]

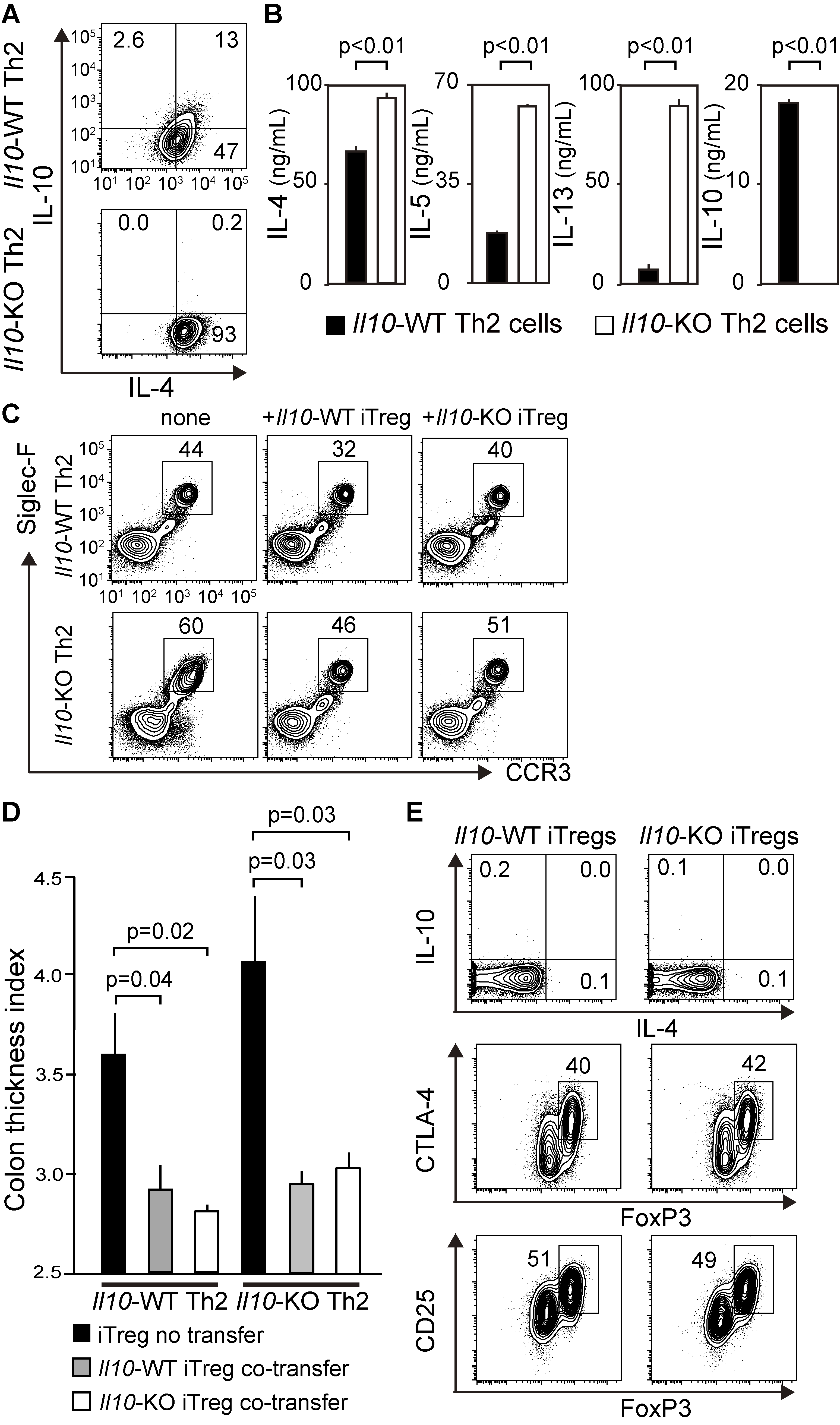

Supplement: S4 Fig — Th2 and iTregs were differentiated in vitro from CD4+ T cells derived from Il10-deficient (Il10-KO:DO11.10+:Rag2-KO) or Il10-sufficient (Il10+/+:DO11.10+:Rag2-KO) mice. (A) Il10-sufficient (Il10-WT) and Il10-deficient (Il10-KO) Th2 cells were restimulated with 12-O-Tetradecanoylphorbol-13-acetate (PMA) (20 ng /mL) and ionomycin (1 μM) in the presence of monensin for 4 h. Cells were stained with mAbs against CD4, and the T cell receptor (DO11.10), and treated with Fixable Viability Dye, and subjected to flow cytometric analysis after incubation with antibodies against IL-4 and IL-10. The frequencies of cells expressing IL-4 or IL-10 were determined according to populations gated on CD4+DO11.10 TCR+ cells. (B) Il10-WT and Il10-KO Th2 cells were restimulated for 48 h in vitro using the anti-CD3epsilon-/anti-CD28-conjugated beads, and secreted cytokines were quantified as described in Materials and Methods. All experiments were reproducibly repeated at least twice, and a representative data set is shown. (C) Il10-WT Th2 cells or Il10-KO Th2 cells along with Il10-sufficient iTregs or Il10-deficient iTregs were transferred to wild-type BALB/c mice and mice. Mononuclear cells (MNCs) were isolated from the cLP of mice engrafted with each combination of cells as indicated and incubated with antibodies against CCR3 and Siglec-F. Representative flow cytometric profiles are shown with the frequencies of CCR3+Siglec-F+ cells in MNCs isolated from the cLP. (D) CTI values were calculated and are shown as mean and standard error (SE). (E) Il10-sufficient (Il10-WT) and Il10-deficient (Il10-KO) iTregs were restimulated as in (A) and stained with anti-CD4, anti-DO11.10 TCR, and anti-CD25 mAbs followed by Fixable Viability Dye staining. Cells were subjected to flow cytometry to determine the intracellular expression of IL-4, IL-10, CTLA4, and FoxP3. CD4+DO11.10 TCR+ FVD− cells were gated for the analysis. (TIF) [file pone.0150244.s004.tif]

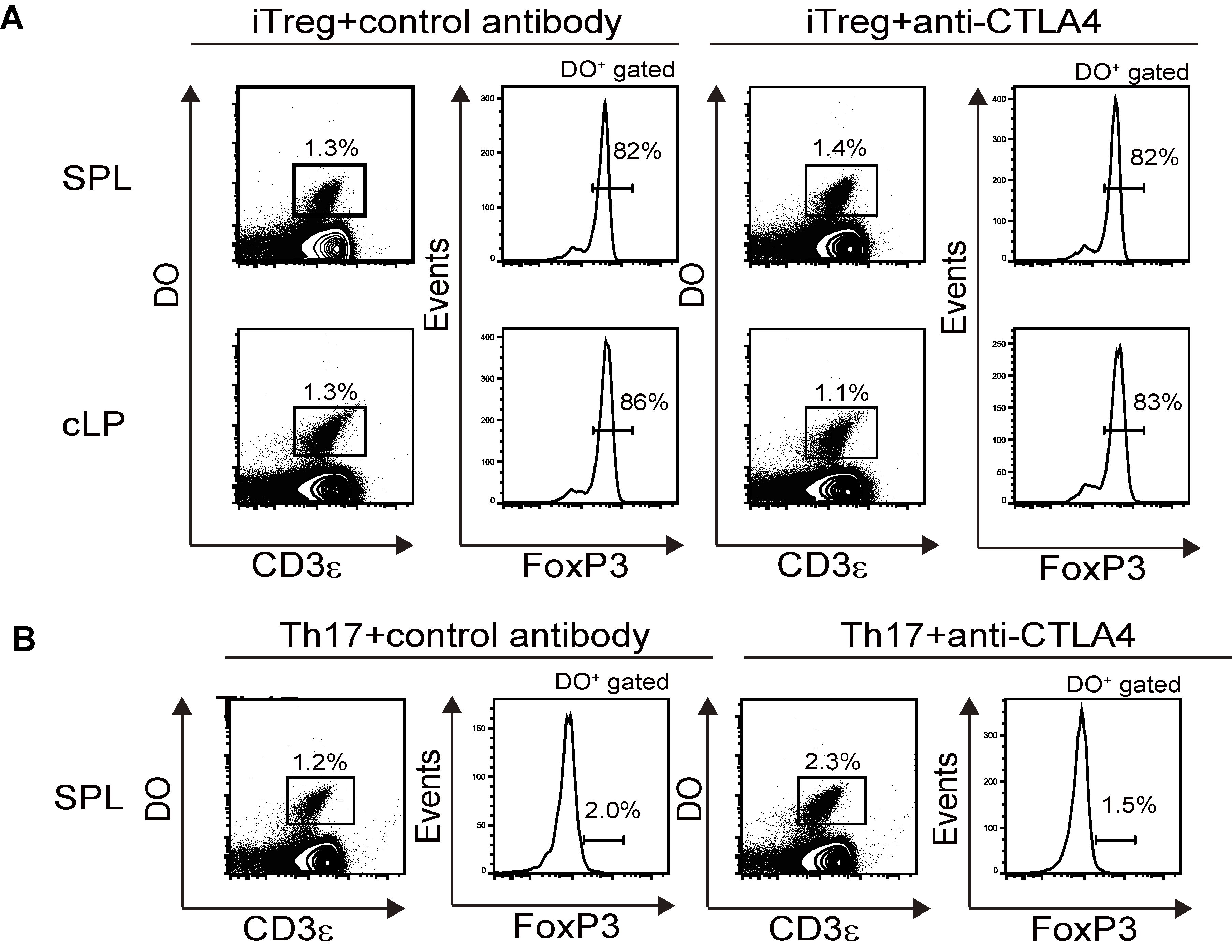

Supplement: S5 Fig — (A) MNCs were isolated from the spleen and cLP of mice engrafted with iTregs in the presence of anti-CTLA4 antibody (20 microg) or control antibody (20 microg) and treated as described in Fig 1. CD4+ T cells were enriched using anti-CD4 magnetic beads (Miltenyi Biotech) and stained with indicated antibodies. Representative flow cytometric profiles are shown with the frequencies of CD3epsilon+DO+ cells and FoxP3+ cells gated on CD3epsilon+DO+ cells. (B) As a control for FoxP3 staining, Th17 cells were used and analyzed in the same way as described in (A). (TIF) [file pone.0150244.s005.tif]

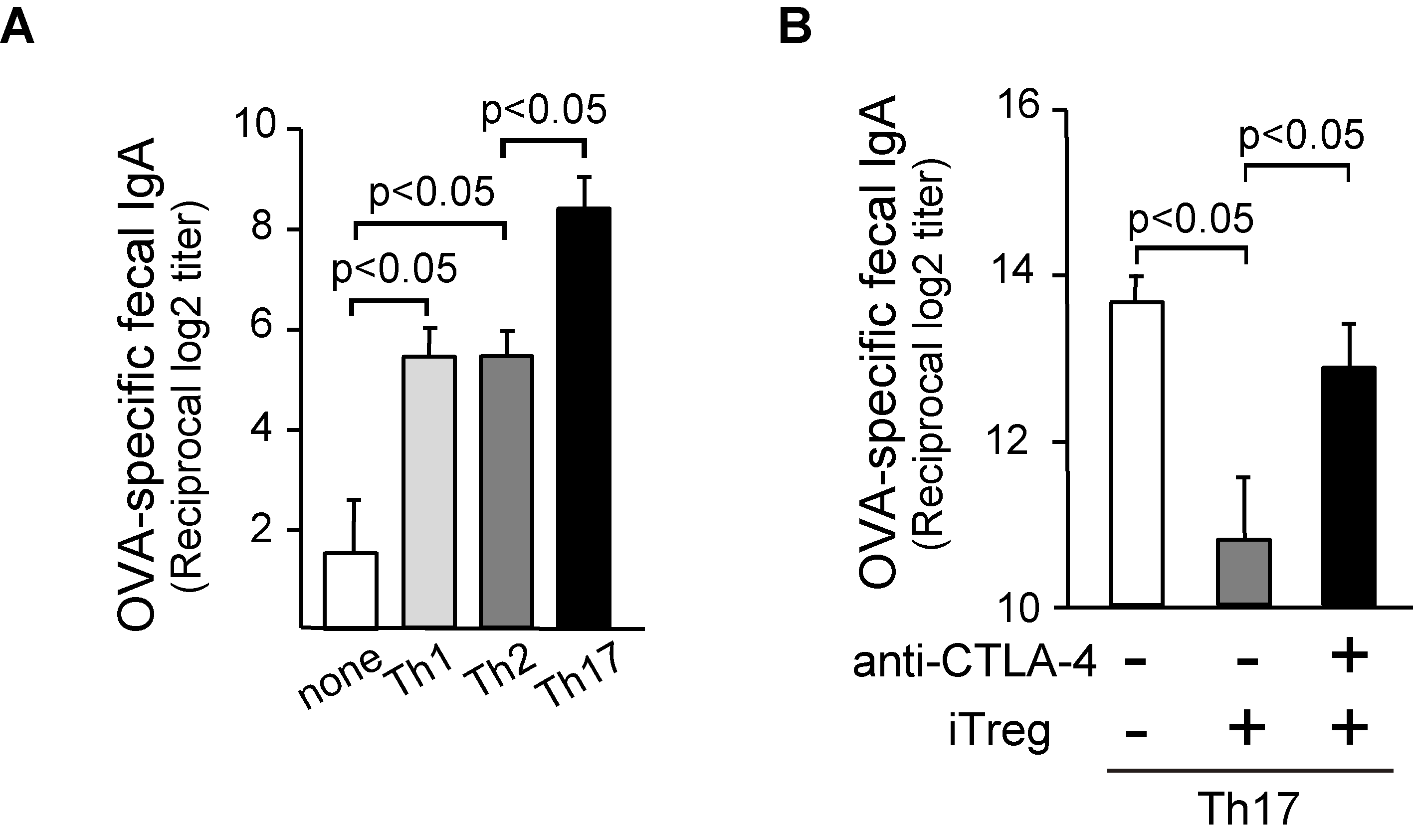

Supplement: S6 Fig — (A) Fecal extracts were prepared the day after the third challenge with OVA, and OVA-specific IgA was detected using ELISA to estimate relative endpoint titers as described in Materials and Methods. (B) Th17 cells with or without iTregs were transferred in the absence or presence of an anti-CTLA4 antibody (20 microg). After the third challenge with OVA, fecal samples were collected, and relative titers of OVA-specific IgA were determined. (TIF) [file pone.0150244.s006.tif]

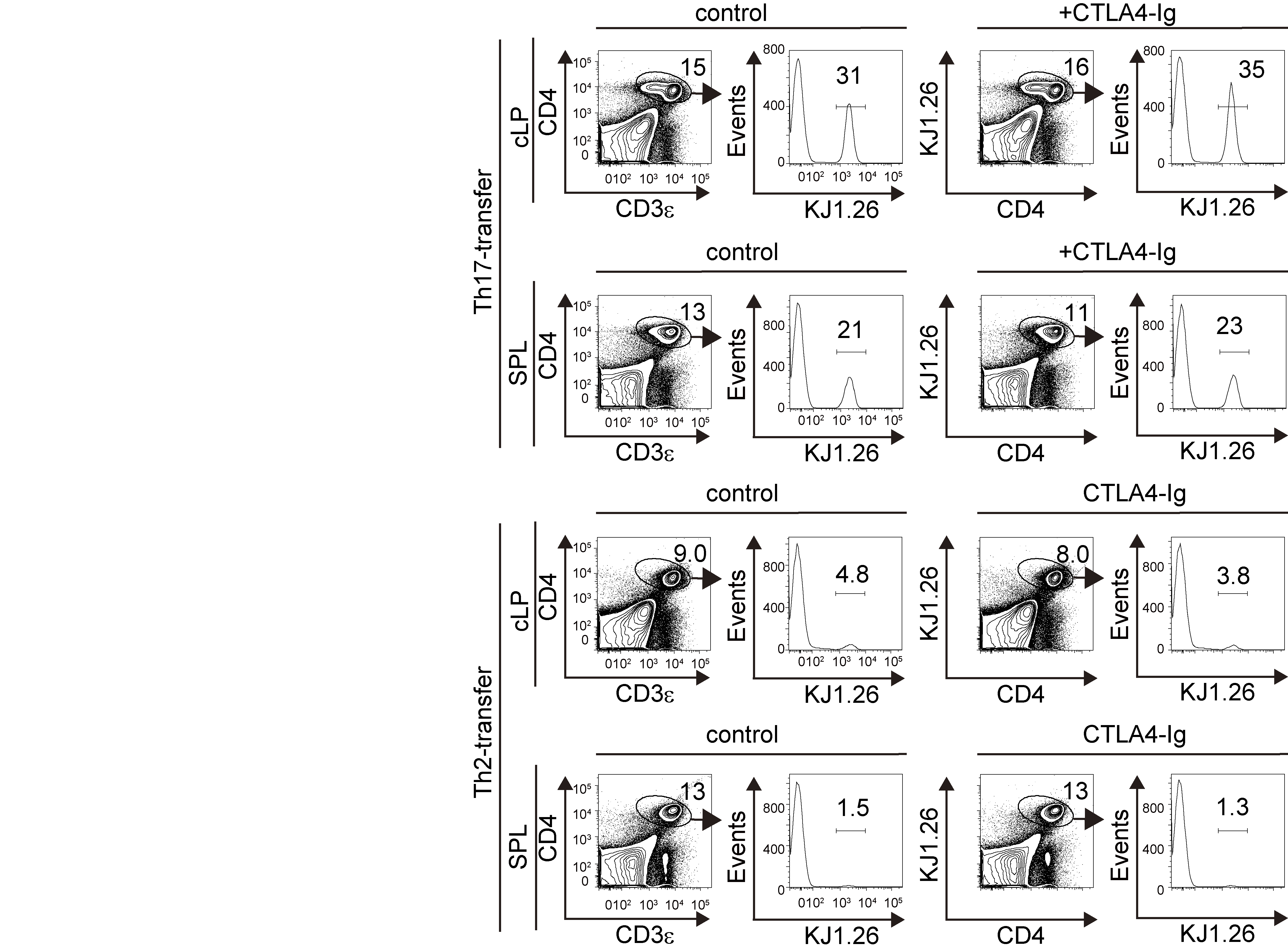

Supplement: S7 Fig — Effector T cells (Th2 or Th17) were intravenously transferred in the presence or absence of CTLA4-Ig (50microg), and mice were treated as described in Fig 1. MNCs were isolated from the SPL or cLP of mice engrafted and subjected to the flow cytometric analysis. Frequencies of CD3epsilon+CD4+ cells were shown and gated populations were analyzed for KJ1.26 staining and ratio of DO (KJ1.26)+ cells were shown in histograms. (TIF) [file pone.0150244.s007.tif]

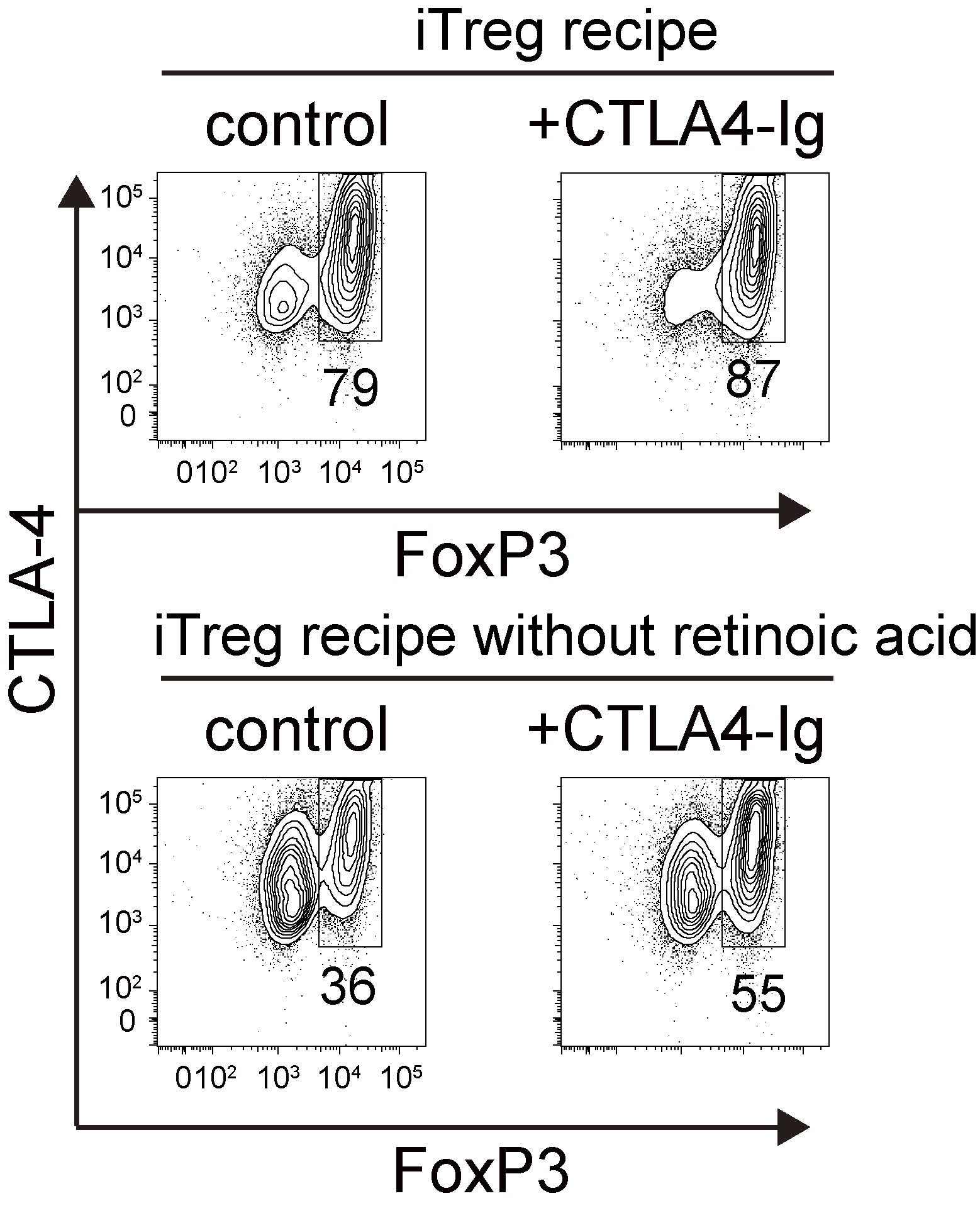

Supplement: S8 Fig — CD4+ T cells prepared from DO11.10+:Rag2-KO mice were stimulated under the condition for iTreg lineage, namely medium supplemented with IL-2, TGF-beta1 and retinoic acid (iTreg recipe), or medium containing IL-2 and TGF-beta1 but not retinoic acid (iTreg recipe without retinoic acid) in the absence (control) or presence of CTLA4-Ig (+CTLA4-Ig, 20microg/mL). After 7 days, cells were subjected to the analysis as described in S1 Fig. (TIF) [file pone.0150244.s008.tif]
